# CCR4 Antagonist (C021) Administration Diminishes Hypersensitivity and Enhances the Analgesic Potency of Morphine and Buprenorphine in a Mouse Model of Neuropathic Pain

**DOI:** 10.3389/fimmu.2020.01241

**Published:** 2020-07-14

**Authors:** Joanna Bogacka, Katarzyna Ciapała, Katarzyna Pawlik, Klaudia Kwiatkowski, Jan Dobrogowski, Anna Przeklasa-Muszynska, Joanna Mika

**Affiliations:** ^1^Department of Pain Pharmacology, Maj Institute of Pharmacology, Polish Academy of Sciences, Krakow, Poland; ^2^Department of Pain Research and Treatment, Chair of Anesthesiology and Intensive Therapy, Jagiellonian University Medical College, Krakow, Poland

**Keywords:** CCL17, CCL22, CCL2, chemokines, opioids, mice, morphine, buprenorphine

## Abstract

Neuropathic pain is a chronic condition that remains a major clinical problem owing to high resistance to available therapy. Recent studies have indicated that chemokine signaling pathways are crucial in the development of painful neuropathy; however, the involvement of CC chemokine receptor 4 (CCR4) has not been fully elucidated thus far. Therefore, the aim of our research was to investigate the role of CCR4 in the development of tactile and thermal hypersensitivity, the effectiveness of morphine/buprenorphine, and opioid-induced tolerance in mice exposed to chronic constriction injury (CCI) of the sciatic nerve. The results of our research demonstrated that a single intrathecal or intraperitoneal administration of C021, a CCR4 antagonist, dose dependently diminished neuropathic pain-related behaviors in CCI-exposed mice. After sciatic nerve injury, the spinal expression of *CCL17* and *CCL22* remained unchanged in contrast to that of *CCL2*, which was significantly upregulated until day 14 after CCI. Importantly, our results provide evidence that in naive mice, CCL2 may evoke pain-related behaviors through CCR4 because its pronociceptive effects are diminished by C021. In CCI-exposed mice, the pharmacological blockade of CCR4 enhanced the analgesic properties of morphine/buprenorphine and delayed the development of morphine-induced tolerance, which was associated with the silencing of IBA-1 activation in cells and decrease in CCL2 production. The obtained data suggest that the pharmacological blockade of CCR4 may be a new potential therapeutic target for neuropathic pain polytherapy.

## Introduction

Neuropathic pain is associated with the dysfunction of or damage to the central or peripheral nervous system. Despite many years of research in this field, the mechanism underlying the generation and persistence of this type of pain is still not fully understood, and this has resulted in a lack of appropriate and effective therapies. This is strictly due to the need for extended treatment, which results in an increased probability of side effects (1). People suffering from neuropathic pain are afflicted by impaired quality of life and continuous visits to health-care providers. The management of painful neuropathy remains challenging because opioids are less effective for neuropathic pain than for other types of pain; therefore, it is necessary to search for new, efficient, and long-acting analgesics (2). It was recently suggested that the strong activation of non-neuronal cells, especially microglia/macrophages, causes decreased opioid-induced analgesic effects in neuropathic pain (3–6). Pronociceptive cytokines (e.g., IL-1beta, IL-18, IL-6, CCL2, and CCL5) released by these cells are considered important factors responsible for reductions in opioid effectiveness (7–11). Importantly, transmembrane cytokine receptors expressed by neurons and microglia/macrophages are essential for cell activation, which makes them promising targets for pharmacological interventions (12). Recent studies have indicated that the blockade of CC chemokine receptors (CCRs), e.g., CCR2 and CCR5, attenuates neuropathic pain symptoms (13–16). Considering that neuronal (17) and non-neuronal cells (18, 19), including human microglia/macrophage (20), have been reported to express CCR4, we hypothesize that the blockade of this receptor may serve as a new target for neuropathic pain pharmacotherapy. CCR4 is a main receptor for CCL17 and CCL22 (18, 21, 22), and its role in nociception has not been studied in detail. It was previously suggested that CCL2 may also act by binding with CCR4 (19). The pronociceptive properties of CCL2 have already been described (23–25).

We hypothesize that CCR4 is important for nociceptive processes in neuropathy; therefore, in the present paper, we investigated the dose-dependent influence of a single intrathecal (i.t.) or intraperitoneal (i.p.) administration of C021, a CCR4 antagonist, on tactile and thermal hypersensitivity in mouse model of neuropathic pain. Additionally, we studied the changes in the spinal expression of CCR4 potential ligands (CCL17, CCL22, and CCL2) in mice following chronic constriction injury (CCI) of the sciatic nerve. Moreover, the aim of this study was to determine how the i.t. administration of C021 influences tactile and thermal hypersensitivity evoked by CCL17, CCL22, or CCL2 injection in naive animals. In a subsequent experiment, we investigated whether a single administration of C021 influences the analgesic effects of morphine and buprenorphine in CCI-exposed rodents. Finally, we examined how repeated (twice daily for 12 days) i.p. injection of C021 impacts the effectiveness of chronically administered morphine and buprenorphine in CCI-exposed mice. Moreover, we revealed changes in the levels of IBA-1 (a microglia/macrophage activation marker), GFAP (an astrocyte/satellite cell activation marker), and some pronociceptive factors (CCL2, IL-1beta, IL-18, and iNOS).

## Materials and Methods

### Animals

Male albino Swiss mice (20–22 g) were purchased from Charles River (Hamburg, Germany) and housed in cages lined with sawdust under a standard 12/12-h light/dark cycle (lights on at 06:00 A.M.). Food and water were available *ad-libitum*. All of the procedures were performed according to the recommendations of the International Association for the Study of Pain (IASP) (26) and the National Institutes of Health (NIH) Guide for the Care and Use of Laboratory Animals. The study protocol was approved by the II Local Ethics Committee branch of the National Ethics Committee for Experiments on Animals based at the Maj Institute of Pharmacology, Polish Academy of Sciences (Krakow, Poland, LKE 75/2017, 1277/2015). Care was taken to minimize animal suffering and reduce the number of animals used in the experiments (3R policy).

### Neuropathic Pain Model

Mice underwent CCI of the sciatic nerve under isoflurane anesthesia according to the procedure described by Bennet and Xie (27). An incision was made below the right hipbone, and the *biceps femoris* and *gluteus superficialis* were separated. The right sciatic nerve was exposed, and three ligatures (4/0 silk sutures) spaced 1 mm apart were tied loosely around the nerve distal to the sciatic notch until a brief twitch was elicited in the respective hind limb. CCI is a standard procedure that has been used in our laboratory for many years to induce neuropathic pain-related behaviors in rodents (15, 25, 28).

### Behavioral Tests

#### Von Frey Test

Tactile hypersensitivity was measured using calibrated nylon monofilaments (0.6–6 g) (Stoelting, Wood Dale, USA) to observe reactions to mechanical stimuli, as described previously (25). The mice were placed in plastic cages with a wire mesh floor 5 min before the experiment, and a von Frey filament was applied to the midplantar surface of the hind paw until the hind paw was lifted. In naive animals, both hind paws were tested in the same way.

#### Cold Plate Test

Thermal hypersensitivity was measured using a cold plate analgesia meter (Ugo Basile, Gemonio, Italy) as described previously (25). The temperature of the cold plate was kept at 2°C. The cutoff latency was 30 s. The animals were placed on the cold plate, and the latency until the hind paw was lifted was recorded. In CCI-exposed mice, the injured foot was the first to react to the cold stimulus in every case. In naive mice, both hind paws were observed simultaneously.

#### Rotarod Test

The rotarod test is a commonly used method to measure motor coordination in animals by assessing their ability to walk on a rotating rod. This test was performed as previously described (29). Mice were placed in a separate compartment on a rotating horizontal rod that accelerated from 2 to 40 rpm within 300 s. The animals were habituated to the apparatus and trained to remain on the rotating rod. The main experiment was performed after training sessions that each lasted 300 s. The rotarod test was conducted 1 h after drug administration. When animals fall from the apparatus, the time was recorded.

### Drug Administration

C021 dihydrochloride (C021; CCR4 antagonist; Tocris, Bristol, UK); morphine hydrochloride [M; μ-opioid receptor (MOR), δ-opioid receptor (DOR), and κ-opioid receptor (KOR) agonist; TEVA, Kutno, Poland]; buprenorphine [B; MOR and nociceptin receptor (NOR) agonist and DOR and KOR antagonist; Polfa S.A., Warsaw, Poland], and recombinant mouse CCL17/CCL22/CCL2 proteins (R&D Systems, Minneapolis, USA) were dissolved in water for injection. The control groups received water for injection (V) on the same schedule. The substances were administered i.t. or i.p. The i.t. injections were performed using a Hamilton syringe with a thin needle in accordance with the literature (30). The substances were injected into the lumbar part of the spinal cord (between the L5 and L6 vertebral space) in a volume of 5 μl. The i.p. injections were performed in accordance with PolLASA (Polish Laboratory Animal Science Association) guidelines.

1. **C021 Administration in Chronic Constriction Injury-Exposed Mice**
  The V or CCR4 antagonist was administered i.t. (10, 20, or 30 μg/5 μl) or i.p. (1, 5, 10, or 20 mg/kg) on day 7 after CCI. Behavioral tests were conducted 1, 4, and 24 h after V or C021 injection. Day 7 was chosen because this time point, in the used model of neuropathy, is considered as a time when animals fully develop thermal and mechanical hypersensitivity, which relatively well-reflects human neuropathic pain symptoms (27, 31–34).
2. Intrathecal CCL Administration Preceded by C021 Injection in Naive Mice
  The animals received a single i.t. injection of V or C021 (30 μg/5 5l). Then, after 15 min, V or recombinant mouse CCL17/CCL22/CCL2 proteins (each 10 ng/5 μl) were administered i.t. The behavioral tests were conducted 1, 4, and 24 h after the injection of the reconstituted chemokines (i.e., 1 h 15 min, 4 h 15 min, and 24 h 15 min after V or C021 administration). The doses of recombinant mouse CCL17/CCL22/CCL2 proteins were chosen based on the dose–response curves published in our previous papers (25, 35).
3. Single Coadministration of C021 and Opioids
  The CCR4 antagonist was administered i.t. (30 μg/5 μl) or i.p. (5 mg/kg) on day 7 after CCI. Then, after 30 min, the V- and C021-treated mice received a single injection of morphine/buprenorphine (i.t., 1 μg/5 μl; i.p., 5 mg/kg). Behavioral assessments were conducted 30 min after opioid administration.
4. Repeated Coadministration of C021 and Opioids
  In CCI-exposed mice, tolerance development was studied administering morphine (30 mg/kg i.p.)/buprenorphine (10 mg/kg i.p.) twice daily with or without C021 for 12 days starting from the first day after CCI. In the experimental groups, C021 (5 mg/kg, i.p.) was administered 16 and 1 h before CCI and twice daily 30 min before each morphine/buprenorphine administration for the following 12 days. The control groups received V+V, C021+V, V+M, or V+B on the same experimental schedule. The behavioral tests were conducted every 2 days 30 min after first morphine/buprenorphine administration.

### Analysis of Gene Expression (RT-qPCR)

Tissue from ipsilateral lumbar segments of the spinal cord (L4–L6) was collected immediately after decapitation from naive and CCI-exposed mice on days 2, 7, and 14 after sciatic nerve injury for quantitative real-time PCR (RT-qPCR) analysis. Total RNA extraction was performed using TRIzol reagent (Invitrogen, Carlsbad, USA) on the basis of a previously described protocol (36). The concentration and quality of RNA were measured by a DeNovix DS-11 Spectrophotometer (DeNovix Inc., Wilmington, USA). The Omniscript RT Kit (Qiagen Inc., Hilden, Germany), oligo (dT16) primer (Qiagen Inc., Hilden, Germany), and RNAse inhibitor (rRNasin, Promega, Mannheim, Germany) were used for reverse transcription of 1 μg of total RNA. The obtained cDNA was diluted 1:10 with RNase-/DNase-free H_2_O. RT-qPCR was conducted with ~50 ng of cDNA from each sample using Assay-On-Demand TaqMan probes (Applied Biosystems, Foster City, USA) on an iCycler device (Bio-Rad, Hercules, Warsaw, Poland). The following TaqMan primers were used: Mm03024075_m1 (hypoxanthine-guanine phosphoribosyltransferase, *HPRT*); Mm01244826_g1 (*CCL17*); Mm00436439_m1 (*CCL22*); and Mm00441243_g1 (*CCL2*). Based on our previous study (25), *HPRT* was used as an endogenous control and an adequate housekeeping gene. There were no significant changes in *HPRT* expression between groups.

### Analysis of Protein Levels (Western Blotting)

Tissue from ipsilateral lumbar segments of the spinal cord (L4–L6) was collected 6 h after the last V/C021 administration on the 12th day of the experiment. The samples were homogenized in radioimmunoprecipitation assay (RIPA) buffer containing a protease inhibitor cocktail (Sigma-Aldrich, St. Louis, USA) and cleared via centrifugation (30 min, 14,000 rpm, 4°C). Total protein concentrations were measured using the bicinchoninic acid (BCA) method. Samples (10 μg of protein) were heated in loading buffer (4 × Laemmli buffer, Bio-Rad, Warsaw, Poland) for 5 min at 98°C. Electrophoresis was performed on 4–15% TGX precast polyacrylamide gels (Bio-Rad, Warsaw, Poland). The proteins from the gels were transferred (semidry transfer, 30 min, 25 V) to Immun-Blot polyvinylidene difluoride (PVDF) membranes (Bio-Rad, Warsaw, Poland). Next, the membranes were blocked for 1 h at room temperature using 5% nonfat dry milk (Bio-Rad, Warsaw, Poland) in Tris-buffered saline with 0.1% Tween-20 (TBST). Then, the membranes were washed in TBST buffer and incubated overnight at 4°C with the following primary antibodies: anti-IBA-1 (1:500, Novus, Abingdon, UK); anti-GFAP (1:10000, Novus, Abingdon, UK); rabbit anti-IL-1beta (1:500, Abcam, Cambridge, UK), anti-IL-18 (1:500, Abcam, Cambridge, UK), anti-iNOS (1:300, Proteintech, Manchester, UK); and mouse anti-GAPDH (1:5,000, Millipore, Darmstadt, Germany). Then, the membranes were washed in TBST buffer and incubated for 1 h at room temperature in horseradish peroxidase (HRP)-conjugated secondary antibodies (Vector Laboratories, Burlingame, USA) at a dilution of 1:5,000. To dilute the primary and secondary antibodies, solution from the SignalBoost™ Immunoreaction Enhancer Kit (Millipore, Darmstadt, Germany) was used. Selected proteins were detected using Clarity™ Western ECL Substrate (Bio-Rad, Warsaw, Poland) and visualized by a Fujifilm LAS-4000 FluorImager system. Fujifilm Multi Gauge software was used to estimate the levels of the immunoreactive bands.

### Analysis of Protein Levels (ELISA)

The CCL2 has low molecular weight; therefore, for detection, the enzyme-linked immunosorbent assay (ELISA) method was used. The samples were homogenized in RIPA buffer containing a protease inhibitor cocktail (Sigma-Aldrich, St. Louis, USA) and cleared via centrifugation (30 min, 14,000 rpm, 4°C). ELISA for CCL2 was performed according to the manufacturer's instructions (mouse CCL2/MCP1 ELISA Kit, LifeSpan Biosciences, Seattle, USA). The detection limit for CCL2 was 15.6–1,000 pg/ml. A positive control for the assay was provided by the manufacturer.

### Data Analysis

The behavioral and biochemical data are presented as the mean ± SEM. To emphasize the overall impact and compare effects from various behavioral tests, the area under the curve (AUC) of antinociceptive effects was calculated. To evaluate the AUCs, trapezoidal and Simpson's rules were used as described by Tallarida and Murray (37). The AUCs (1–12 days) were calculated from the observed values (seconds for the cold plate test and grams for the von Frey test). One-way analysis of variance (ANOVA) was applied to evaluate the experimental results, and Bonferroni's *post-hoc* test was used to analyze the differences between the groups. Additionally, some results were evaluated using two-way ANOVA to determine the time × drug interaction.

## Results

### The Influence of Single Intrathecal or Intraperitoneal C021 Administration on Neuropathic Pain-Related Behaviors Measured on the Seventh Day After Chronic Constriction Injury

**Figure 1:**
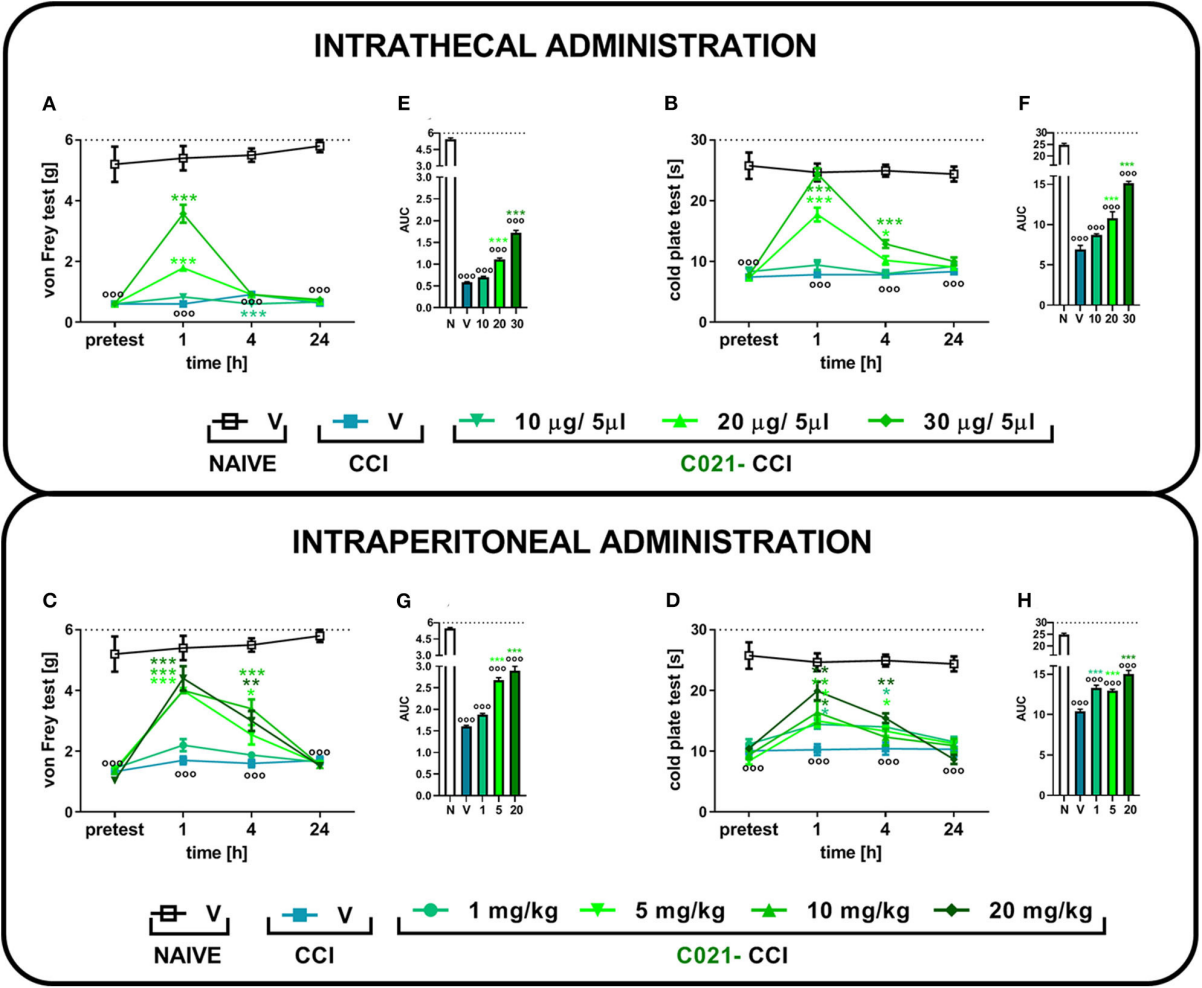
The intrathecal or intraperitoneal administration of C021 (a CCR4 antagonist) dose dependently diminished hypersensitivity in CCI-exposed mice, as measured by the von Frey and cold plate tests. The effects of a single i.t. (10, 20, or 30 μg/5 μl; **A,B**) or i.p. (1, 5, 10, or 20 mg/kg; **C,D**) injection of C021 7 days after chronic constriction injury of the sciatic nerve on tactile (von Frey test; **A,C**) and thermal (cold plate test; **B,D**) hypersensitivity measured 1, 4, and 24 h following antagonist administration. The data are presented as the mean ± SEM and the numbers of animals (shown as a *n* = mice tested in von Frey/*n* = mice tested in cold plate) are as follows: i.t. N (*n* = 5/5) and CCI, V (*n* = 6/6), 10 (*n* = 6/6), 20 (*n* = 7/7), and 30 (*n* = 7/7); i.p. N (*n* = 5/5) and CCI, V (*n* = 6/6), 1 (*n* = 10/10), 5 (*n* = 10/10), 10 (*n* = 10/9), and 20 (*n* = 10/7). Additionally, the data obtained from von Frey **(A,C)** and cold plate **(B,D)** tests were analyzed as areas under the curve (AUCs, **E–H**). The results were evaluated using one-way ANOVA followed by Bonferroni's multiple comparisons *post hoc* test of selected pairs measured separately at each time point. ^ooo^*p* < 0.001 indicates differences between naive and CCI-exposed mice **(A–D)** and all groups compared with naive mice **(E–H)**. *p < 0.05, **p < 0.01, and ***p < 0.001 indicate significant differences between the V-treated and C021-treated CCI-exposed animals. Abbreviations: C021, C021 dihydrochloride; CCI, chronic constriction injury; N, naive, V, vehicle. The dotted lines indicate the cutoff value for the tests.

Sciatic nerve injury evokes strong tactile and thermal hypersensitivity in mice (*p* < 0.001, Figure 1). The i.t. (Figures 1A,B) and i.p. (Figures 1C,D) C021 administration dose dependently diminished tactile and thermal hypersensitivity 1, 4, and 24 h after treatment. In the von Frey test, the i.t. administration of C021 at a dose of 30 μg/5 μl had the most robust effect, significantly [*F*_(4, 27)_ = 83.84, *p* < 0.0001] attenuating tactile hypersensitivity 1 h after injection (Figure 1A). The i.p. administration of C021 at doses of 5, 10, and 20 mg/kg caused similar analgesic effects 1 h after administration (Figure 1C). The observed effects lasted until 4 h after C021 administration. In the cold plate test, the i.t. administration of C021 (30 μg/5 μl), compared with that of V, the most significantly reduced thermal hypersensitivity [*F*_(4, 25)_ = 69, *p* < 0.0001] 1 h after injection (Figure 1B). The i.p. dose that had the most robust effect on performance in the cold plate test was 20 mg/kg, which led to the attenuation of thermal hypersensitivity [*F*_(5, 41)_ = 10.68, *p* < 0.0001] 1 h after injection (Figure 1D). Analysis of the AUCs of the data obtained from the behavioral tests confirmed that the i.t. (Figures 1E,F) and i.p. (Figures 1G,H) administration of C021 to CCI-exposed mice diminished the pain-related symptoms (Figures 1G,H).

### Time-Dependent Study in the Spinal mRNA Levels of *CCL17, CCL22*, and *CCL2* in Chronic Constriction Injury-Exposed Mice

**Figure 2:**
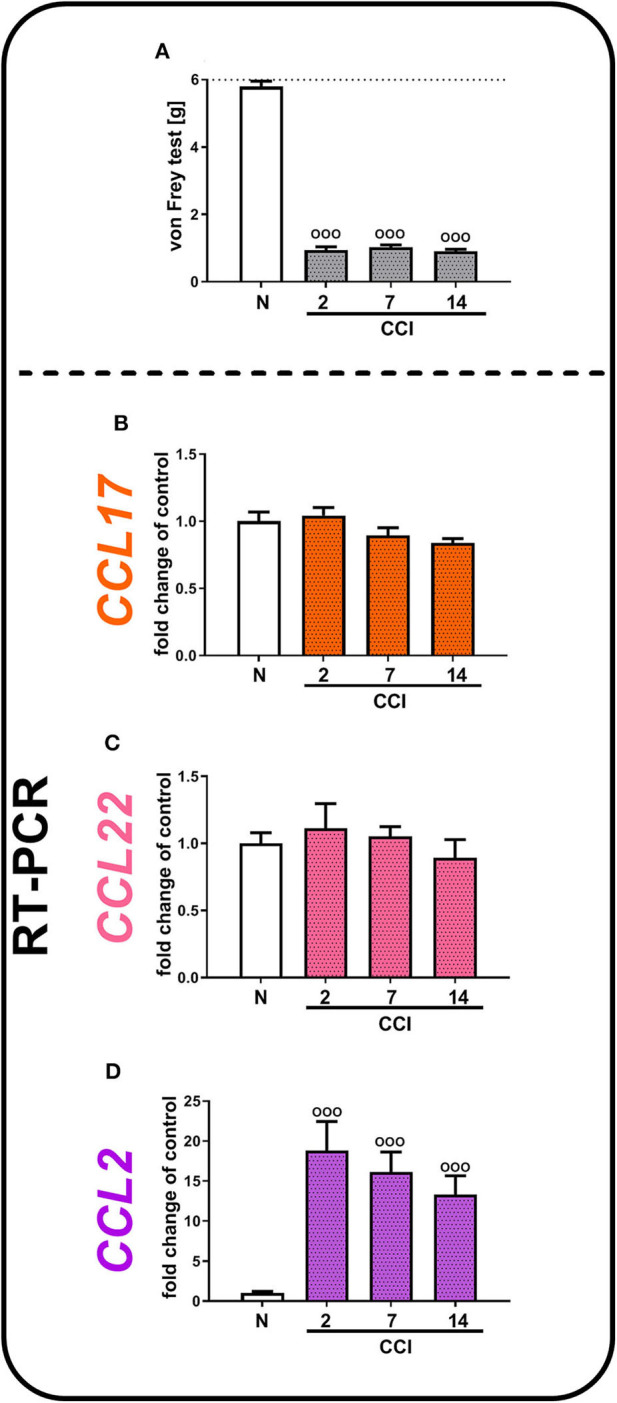
Neuropathic pain-related behaviors **(A)** appear in parallel with spinal changes in the mRNA levels of *CCL2* **(D)**, but not *CCL17* **(B)**, or *CCL22* **(C)**, in (CCI)-exposed mice. Behavioral hypersensitivity was measured by the von Frey test **(A)** 2, 7, and 14 days after injury. The behavioral data are presented as the mean ± SEM; the numbers of animals are as follows: N (*n* = 10) and CCI, 2 days (*n* = 10), 7 days (*n* = 10), and 14 days (*n* = 10). The RT-qPCR data are presented as the fold change relative to control (naive) mice ± SEM; the numbers of samples are as follows: N (*n* = 8–10) and CCI, 2 days (*n* = 8), 7 days (*n* = 7–10), and 14 days (*n* = 8–9). The results were evaluated using one-way ANOVA followed by Bonferroni's multiple comparisons *post hoc* test of selected pairs. ^ooo^*p* < 0.001 indicates differences compared with naive mice. Abbreviations: C021, C021 dihydrochloride; CCI, chronic constriction injury; N, naive; V, vehicle. The dotted line indicate the cutoff value for the test.

In the von Frey test, we observed the development of strong tactile hypersensitivity from the second to 14th days after CCI (Figure 2A). In parallel, we did not observe any significant changes in *CCL17* (Figure 2B) and *CCL22* (Figure 2C) mRNA levels from the second to 14th days after CCI, which remained at a constant low level of expression, compared with that of naive mice. Nevertheless, we demonstrated that the *CCL2* level increased almost 19-fold 2 days after CCI [*F*_(3, 33)_ = 11.37, *p* < 0.0001] and 16-fold 7 days after CCI and then gradually decreased; however, it was still significantly upregulated on day 14 (Figure 2D).

### Effects of a Single Intrathecal Administration of Recombinant CCL17, CCL22, and CCL2 Proteins Preceded by C021 Injection in Naive Mice

**Figure 3:**
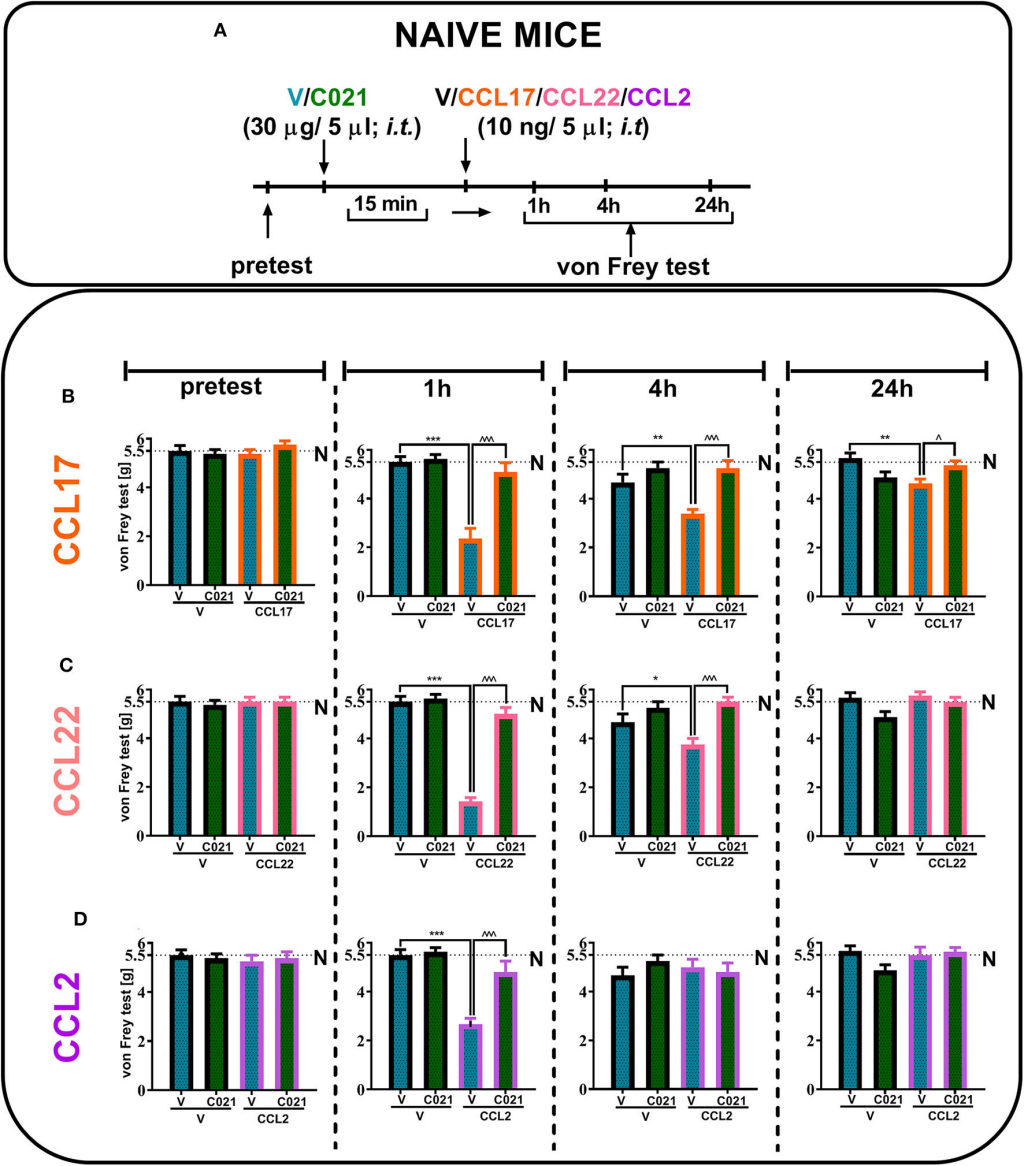
The pronociceptive effect of a single intrathecal administration (10 ng/5 μl) of CCL17 **(B)**, CCL22 **(C)**, or CCL2 **(D)** is diminished by intrathecal pretreatment with C021 (30 μg/5 μl) in naive mice. Behavioral tests were performed 1 h 15 min, 4 h 15 min, and 24 h 15 min after C021 treatment; or 1, 4, and 24 h after CCL administration **(A)**. Tactile hypersensitivity (von Frey test) was measured **(B–D)**. The data are presented as the mean ± SEM, the numbers of animals are as follows: **N** (*n* = 5) and CCI, V+V (*n* = 6), V+CCL17 (*n* = 8), V+CCL22 (*n* = 8), V+CCL2 (*n* = 7), C021+V (*n* = 8), C021+CCL17 (*n* = 8), C021+CCL22 (*n* = 8), and C021+CCL2 (*n* = 7–8); and the results were evaluated by one-way ANOVA followed by Bonferroni's multiple comparisons *post hoc* test of selected pairs at respective time points. *p < 0.05, **p < 0.01, and ***p < 0.001 indicate differences between the V+V-treated and V+CCL17/CCL22/CCL2-treated naive mice; ∧*p* < 0.05 and ∧∧∧*p* < 0.001 indicate differences between the V+CCL17/CCL22/CCL2-treated and C021+CCL17/CCL22/CCL2-treated naive mice. The dotted lines indicate the naive value for the tests. Abbreviations: C021, C021 dihydrochloride; N, naive; V, vehicle.

Our results indicated that a single i.t. administration of V and C021 (30 μg/5 μl) had no effect on tactile hypersensitivity at any of the examined time points, as measured by the von Frey test (Figures 3B–D). Additionally, the results presented as line graphs are in the Supplementary Materials (Supplementary Figure 1). We observed strong tactile hypersensitivity at 1 h after the administration of CCL17 [*F*_3,26_ = 22.19, *p* < 0.0001] (Figure 3B), CCL22 [*F*_(3, 26)_ = 91.63, *p* < 0.0001] (Figure 3C), or CCL2 [*F*_3,24_ = 22.82, *p* < 0.0001] (Figure 3D) (each 10 ng/5 μl), and this effect was no longer observed after 24 h, except in the case of CCL17 administration [*F*_3,26_ = 5.164, *p* = 0.0062]. C021 (30 μg/5 μl) administered 15 min before the i.t. injection of recombinant CCL proteins prevented chemokine-induced hypersensitivity in all examined cases (Figures 3B–D).

### Influence of a Single Intrathecal Administration of C021 on the Effectiveness of Morphine and Buprenorphine on the Seventh Day After Chronic Constriction Injury

**Figure 4:**
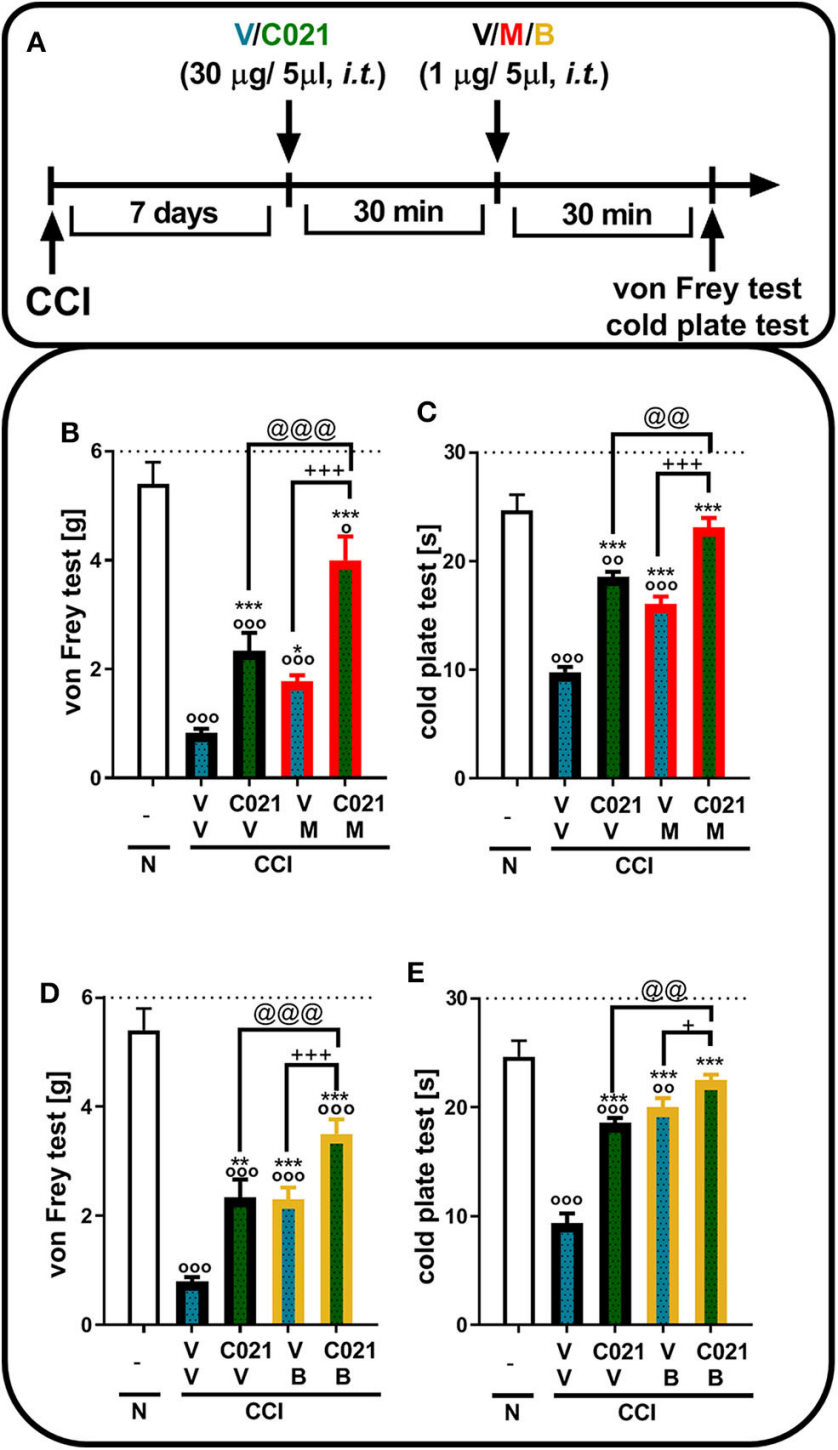
A single intrathecal administration of C021 (CCR4 antagonist; 30 μg/5 μl) enhanced the effectiveness of morphine (1 μg/5 μl) and buprenorphine (1 μg/5 μl) in CCI-exposed mice, as measured by the von Frey **(B,D)** and cold plate **(C,E)** tests. Mice treated with a single administration of V or C021 received a single dose of V, M, or B after 30 min; and 30 min later, behavioral tests were conducted **(A)**. The data are presented as the mean ± SEM and the numbers of animals (shown as a *n* = mice tested in von Frey/*n* = mice tested in cold plate) are as follows: N (*n* = 5/5) and CCI, V+V (*n* = 7/6), C021+V (*n* = 7/7), V+M (*n* = 8/5), C021+M (*n* = 7/4), V+B (*n* = 8/7), and C021+B (*n* = 8/7). The results were analyzed using one-way ANOVA with Bonferroni's multiple comparisons *post hoc* test. ^o^*p* < 0.05, ^oo^*p* < 0.01, ^ooo^*p* < 0.001 indicate differences compared with naive mice; *p < 0.05, **p < 0.01, and ***p < 0.001 compared with V+V-treated CCI-exposed mice; ^+^*p* < 0.05 and ^+++^*p* < 0.001 indicate differences between the V+M/B-treated and C021+M/B-treated CCI-exposed mice; @@*p* < 0.01 and @@@*p* < 0.001 indicate differences between the C021+V-treated and C021+M/B-treated CCI-exposed mice. The dotted lines indicate the cutoff value for the tests. Abbreviations: B, buprenorphine; C021, C021 dihydrochloride; CCI, chronic constriction injury; M, morphine; N, naive, V, vehicle.

Tactile and thermal hypersensitivity was observed in the von Frey and cold plate tests 7 days after CCI (*p* < 0.0001, Figure 4). A single i.t. injection of C021 (30 μg/5 μl), compared with that of V, significantly attenuated CCI-induced tactile hypersensitivity, as measured by the von Frey test (Figures 4B,D). Similarly, in the cold plate test, we observed a reduction in thermal hypersensitivity in C021-treated mice (Figures 4C,E). Tactile and thermal hypersensitivity were also significantly reduced after a single i.t. administration of morphine (1 μg/5 μl) [*F*_4,29_ = 39.53, *p* < 0.0001] or buprenorphine (1 μg/5 μl) [*F*_(4.31)_ = 43.03, *p* < 0.0001], as measured by the von Frey test (Figures 4B,D) and the cold plate test [morphine: *F*_(4.22)_ = 58.43, *p* < 0.0001; buprenorphine: *F*_(4.25)_ = 47.49, *p* < 0.0001] (Figures 4C,E). We observed that pretreatment with C021 potentiated the effects of morphine in the von Frey test [*F*_(4.29)_ = 39.53, *p* < 0.0001] and cold plate test [*F*_(4.22)_ = 58.43, *p* < 0.0001] (Figures 4B,C) and increased buprenorphine-induced analgesia in both the von Frey test [*F*_(4.31)_ = 43.03, *p* < 0.0001] and cold plate test [*F*_(4.25)_ = 47.49, *p* < 0.0001] (Figures 4D,E).

### Influence of a Single Intraperitoneal Administration of C021 on the Effectiveness of Morphine and Buprenorphine on the Seventh Day After Chronic Constriction Injury

**Figure 5:**
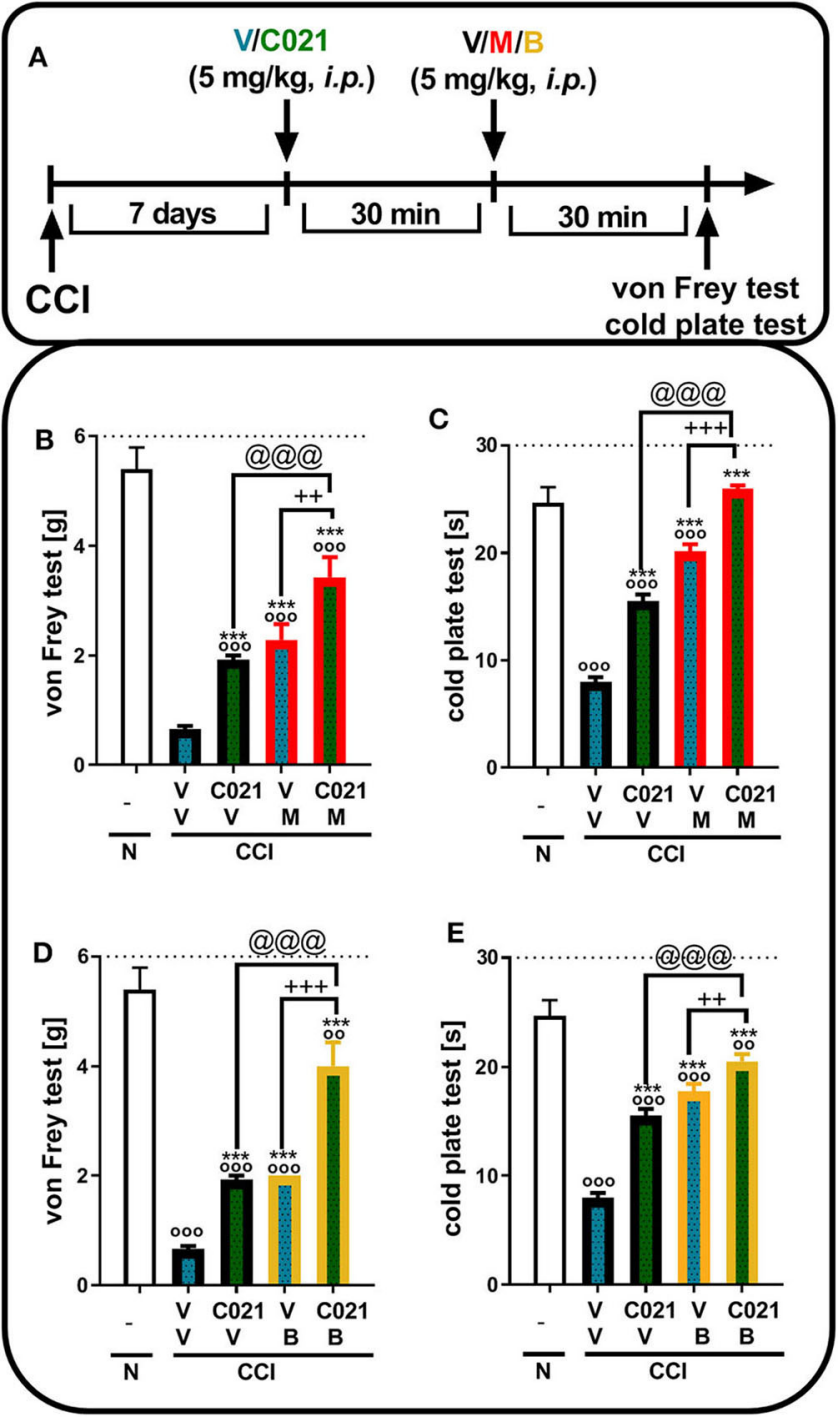
A single intraperitoneal administration of C021 (a CCR4 antagonist; 5 mg/kg) enhanced the effectiveness of morphine (5 mg/kg) and buprenorphine (5 mg/kg) in CCI-exposed mice, as measured by the von Frey **(B,D)** and cold plate **(C,E)** tests. Mice treated with a single administration of V or C021 received a single dose of V, M, or B after 30 min; and 30 min later, behavioral tests were conducted **(A)**. The data are presented as the mean ± SEM, and the numbers of animals (shown as a *n* = mice tested in von Frey/*n* = mice tested in cold plate) are as follows: N (*n* = 5/5) and CCI: V+V (*n* = 7/7), C021+V (*n* = 8/8), V+M (*n* = 7/7), C021+M (*n* = 7/7), V+B (*n* = 7/7), and C021+B (*n* = 8/8). The results were analyzed using one-way ANOVA with Bonferroni's multiple comparisons *post hoc* test. ^oo^*p* < 0.01, ^ooo^*p* < 0.001 indicate differences compared with naive mice; ***p < 0.001 compared with V+V-treated CCI-exposed mice; ^++^*p* < 0.01 and ^+++^*p* < 0.001 indicate differences between the V+M/B-treated and C021+M/B-treated CCI-exposed mice; @@@*p* < 0.001 indicates differences between the C021+V-treated and C021+M/B-treated CCI-exposed mice. Abbreviations: B, buprenorphine; C021, C021 dihydrochloride; CCI, chronic constriction injury; M, morphine; N, naive, V, vehicle. The dotted lines indicate the cutoff value for the tests.

Tactile and thermal hypersensitivity was observed in the von Frey and cold plate tests 7 days after CCI (*p* < 0.0001, Figure 5). A single i.p. injection of C021 (5 mg/kg), compared with that of V, significantly attenuated CCI-induced tactile hypersensitivity 1 h after administration, as measured by the von Frey test (Figures 5B,D); likewise, a reduction in thermal hypersensitivity in the cold plate test (Figures 5C,E) was observed. Hypersensitivity was also significantly diminished after a single i.p. administration of morphine (5 mg/kg) or buprenorphine (5 mg/kg), as measured by the von Frey test [morphine: *F*_(3, 25)_ = 23.79, *p* < 0.0001; buprenorphine: *F*_(3, 25)_ = 39.55, *p* < 0.0001] (Figures 5B,D) and the cold plate test [morphine: *F*_(3, 25)_ = 194.5, *p* < 0.0001; buprenorphine: *F*_(3, 26)_ = 72.16, *p* < 0.0001] (Figures 5C,E). The i.p. administration of C021 enhanced the effectiveness of both morphine and buprenorphine in the von Frey test [morphine: *F*_3,25_ = 23.79, *p* < 0.0001; buprenorphine: *F*_(3, 25)_ = 39.55, *p* < 0.0001] (Figures 5B,D) and cold plate test [morphine: *F*_(3, 25)_ = 194.5, *p* < 0.0001; buprenorphine: *F*_(3, 26)_ = 72.16, *p* < 0.0001] (Figures 5C,E).

### Influence of Repeated Intraperitoneal Administrations of C021 on the Analgesic Effects of Chronic Treatment With Morphine in Chronic Constriction Injury-Exposed Mice

**Figure 6:**
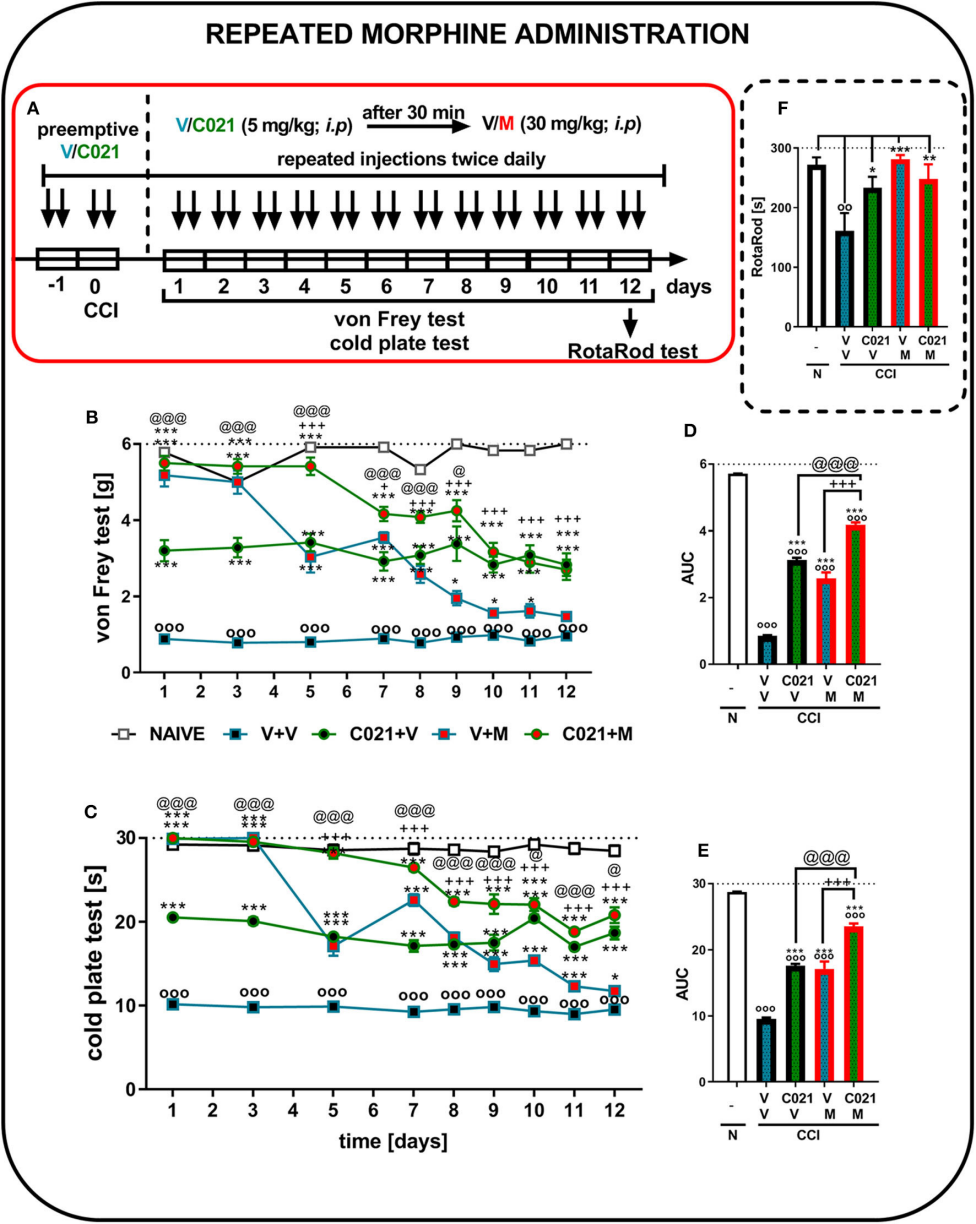
Repeated twice daily intraperitoneal administration of C021 (CCR4 antagonist; 5 mg/kg) attenuated the development of morphine tolerance, as measured by the von Frey **(B,D)** and cold plate **(C,E)** tests, and beneficially influenced motor coordination in CCI-exposed mice, as measured by the rotarod **(F)** test. V and C021 were repeatedly administered i.p. 16 and 1 h before CCI and then twice daily for 12 days 30 min before treatment with V or morphine (M, 30 mg/kg); and 30 min later, the behavioral tests were conducted **(A)**. The data are presented as the mean ± SEM, and the numbers of animals (shown as *n* = mice tested in von Frey/*n* = mice tested in cold plate) are as follows: N (*n* = 6/6) and CCI, V+V (*n* = 12/12), C021+V (*n* = 12/12), V+M (*n* = 11/11), and C021+M (*n* = 12/12). The results were analyzed using one-way ANOVA with Bonferroni's multiple comparisons *post hoc* test. Additionally, the results were evaluated using two-way ANOVA to determine the time × drug interaction (*Result* part, page 10). Additionally, the data obtained from von Frey **(D)** and cold plate **(E)** tests were analyzed as areas under the curve (AUCs). ^oo^*p* < 0.01, ^ooo^*p* < 0.001 indicate differences between naive and CCI-exposed mice **(B,C,F)** and all groups compared with naive mice **(D,E)**; *p < 0.05, **p < 0.01, and ***p < 0.001 indicate differences compared with V+V-treated CCI-exposed mice; +*p* < 0.05 and + + +*p* < 0.001 indicate differences between the V+M-treated and C021+M-treated CCI-exposed mice; @*p* < 0.05 and @@@*p* < 0.001 indicate differences between the C021+V-treated and C021+M-treated CCI-exposed mice. Abbreviations: C021, C021 dihydrochloride; CCI, chronic constriction injury; M, morphine; N, naive, V, vehicle. The dotted lines indicate the cutoff value for the tests.

The behavioral results from naive and vehicle-treated CCI-exposed mice clearly show that neuropathic animals' reactivity to mechanical and thermal stimuli is on a stable significant lower level till 12 days (*p* < 0.0001; Figures 6B,C). Repeated morphine treatment (twice daily; 30 mg/kg, i.p.) for 12 days (Figure 6A) led to the development of morphine tolerance in CCI-exposed mice, as measured by the von Frey test (Figure 6B). The analgesic effect of morphine on day 5 was reduced compared with the effect of morphine on the first day of the experiment; however, it was still significantly stronger than that of V (Figure 6B). Pretreatment with C021 followed by the 12-day administration of C021 (twice daily, 5 mg/kg, i.p.) plus morphine (twice daily, 30 mg/kg, i.p.) significantly improved the analgesic effects of this opioid, as demonstrated by the von Frey test (Figure 6B). Additionally, the injection of C021 alone attenuated the development of tactile hypersensitivity in mice following CCI (Figure 6B). Similar effects were observed in the cold plate test, in which the analgesic effect of morphine on day 5 was reduced compared with to the effect of morphine on the first day of the experiment; however, it was still significantly stronger than that of V (Figure 6C). Pretreatment with C021 followed by the 12-day coadministration of C021 and morphine improved the analgesic effects of morphine, as demonstrated by the cold plate test (Figure 6C). Two-way ANOVA confirmed a significant interaction between the investigated treatment and the considered time points in the von Frey test [*F*_(32, 432)_ = 11, *p* < 0.0001] and cold plate test [*F*_(32, 431)_ = 28.62, *p* < 0.0001]. Analysis of the AUCs of the data obtained from behavioral tests confirmed that the repeated coadministration of C021 and morphine to CCI-exposed mice strongly enhanced the effectiveness of morphine (Figures 6D,E). In parallel, we performed the rotarod test to evaluate changes in the motor activity of the animals after 12 days of administration (Figure 6F). The results of our study revealed that the repeated administration of C021, morphine, and C021 combined with morphine improved the motor coordination of animals, whereas V-treated mice exhibited significant impairments in motor coordination [*F*_(4, 54)_ = 5.384, *p* = 0.0010] (Figure 6F).

### Influence of Repeated Intraperitoneal Administration of C021 on the Analgesic Effects of Chronic Treatment With Buprenorphine in Chronic Constriction Injury-Exposed Mice

**Figure 7:**
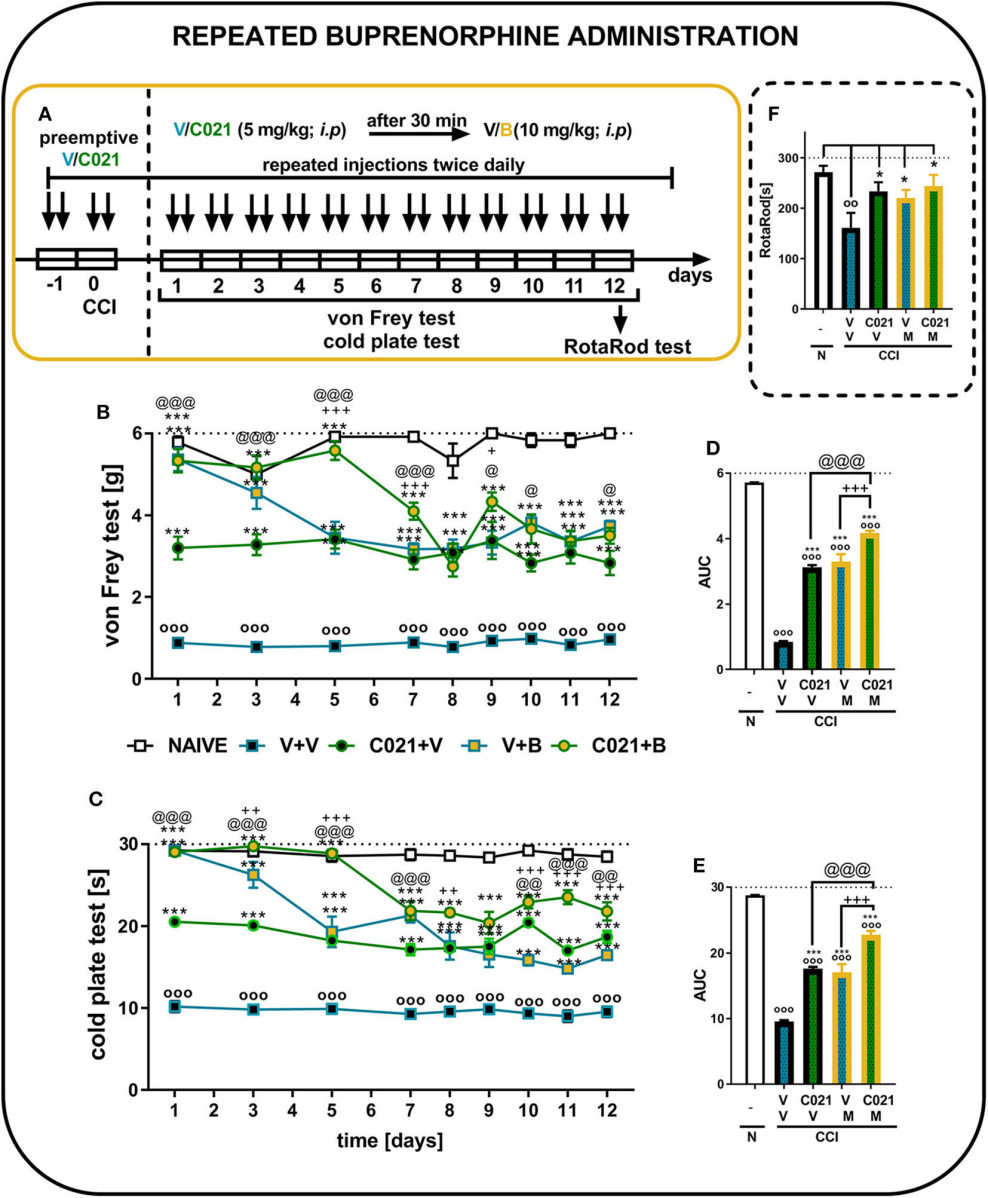
Repeated twice daily intraperitoneal administration of C021 (a CCR4 antagonist; 5 mg/kg) enhanced the analgesic properties of buprenorphine, as measured by the von Frey **(B,D)** and cold plate **(C,E)** tests, and beneficially influenced motor coordination in CCI-exposed mice, as measured by the rotarod **(F)** test. V and C021 were repeatedly administered i.p. 16 and 1 h before CCI and then twice daily for 12 days 30 min before treatment with V or buprenorphine (B, 10 mg/kg); and 30 min later, the behavioral tests were conducted **(A)**. The data are presented as the mean ± SEM, and the numbers of animals (shown as *n* = mice tested in von Frey/*n* = mice tested in cold plate) are as follows: N (*n* = 6/6) and CCI, V+V (*n* = 12/12), C021+V (*n* = 12/12), V+B (*n* = 11/11), and C021+B (*n* = 12/12). The results were analyzed using one-way ANOVA with Bonferroni's multiple comparisons *post hoc* test. Additionally, the results were evaluated using two-way ANOVA to determine the time × drug interaction (Result part, page 10). Additionally, the data obtained from von Frey **(D)** and cold plate **(E)** tests were analyzed as areas under the curve (AUCs). ^oo^*p* < 0.01, ^ooo^*p* < 0.001 indicate differences between naive and CCI-exposed mice **(B,C,F)** and all groups compared with naive mice **(D,E)**; *p < 0.05 and ***p < 0.001 indicate differences compared with V+V-treated CCI-exposed mice; +*p* < 0.05, ++*p* < 0.01, and + + +*p* < 0.001 indicate differences between the V+B-treated and C021+B-treated CCI-exposed mice; @*p* < 0.05, @@*p* < 0.01, and @@@*p* < 0.001 indicate differences between the C021+V-treated and C021+B-treated CCI-exposed mice. Abbreviations: B, buprenorphine; C021, C021 dihydrochloride; CCI, chronic constriction injury; N, naive, V, vehicle. The dotted lines indicate the cutoff value for the tests.

The behavioral results from naive and vehicle-treated CCI-exposed mice clearly show that neuropathic animals' reactivity to mechanical and thermal stimuli is on a stable significant lower level till 12 days (*p* < 0.0001; Figures 7B,C). Repeated buprenorphine treatment (twice daily, 10 mg/kg i.p.) for 12 days did not lead to the development of tolerance; however, a decrease in the analgesic potency of buprenorphine was observed on day 5, as measured by the von Frey test (Figure 7B). Interestingly, pretreatment with and repeated administrations of C021 (twice daily, 5 mg/kg, i.p.) delayed the decrease in the effectiveness of buprenorphine. The antinociceptive effects of buprenorphine C021 and the coadministration of these substances were similar from day 8 until the end of the experiment on day 12, and these effects were significantly stronger than the effect of V, as assessed by the von Frey test (Figure 7B). Similar effects were observed in the cold plate test, in which the analgesic effect of buprenorphine on day 5 was reduced compared with the effect of buprenorphine on the first day; however, the effect was still significantly higher than that of V (Figure 7C). Pretreatment with C021 followed by the 12-day administration of C021 (twice daily; 30 mg/kg, i.p.) combined with buprenorphine significantly improved the analgesic effects of buprenorphine, as demonstrated by the von Frey and cold plate tests (Figures 7B,C). Two-way ANOVA confirmed a significant interaction between the investigated treatment and the investigated time points in the von Frey test [*F*_(32, 430)_ = 4.951, *p* < 0.001] and cold plate test [*F*_(32, 433)_ = 8,998, *p* < 0.001]. Analysis of the AUCs of the data obtained from the behavioral tests also showed that the repeated administration of C021 together with buprenorphine to CCI-exposed mice strongly influenced the analgesic properties of buprenorphine (Figures 7D,E). Simultaneously, we conducted the rotarod test to evaluate changes in the motor activity of animals after 12 days of administration (Figure 7F). The results of our study showed that the repeated administration of C021, buprenorphine, and C021 combined with buprenorphine improved the motor coordination of animals, whereas V-treated mice exhibited significant impairments in motor coordination [*F*_4,55_ = 3.951, *p* = 0.0069] (Figure 7F).

### Influence of the Repeated Intraperitoneal Administration of C021 on the Protein Levels of IBA-1, GFAP, IL-1beta, IL-18, iNOS, and CCL2 in Chronic Constriction Injury-Exposed Mice

**Figure 8:**
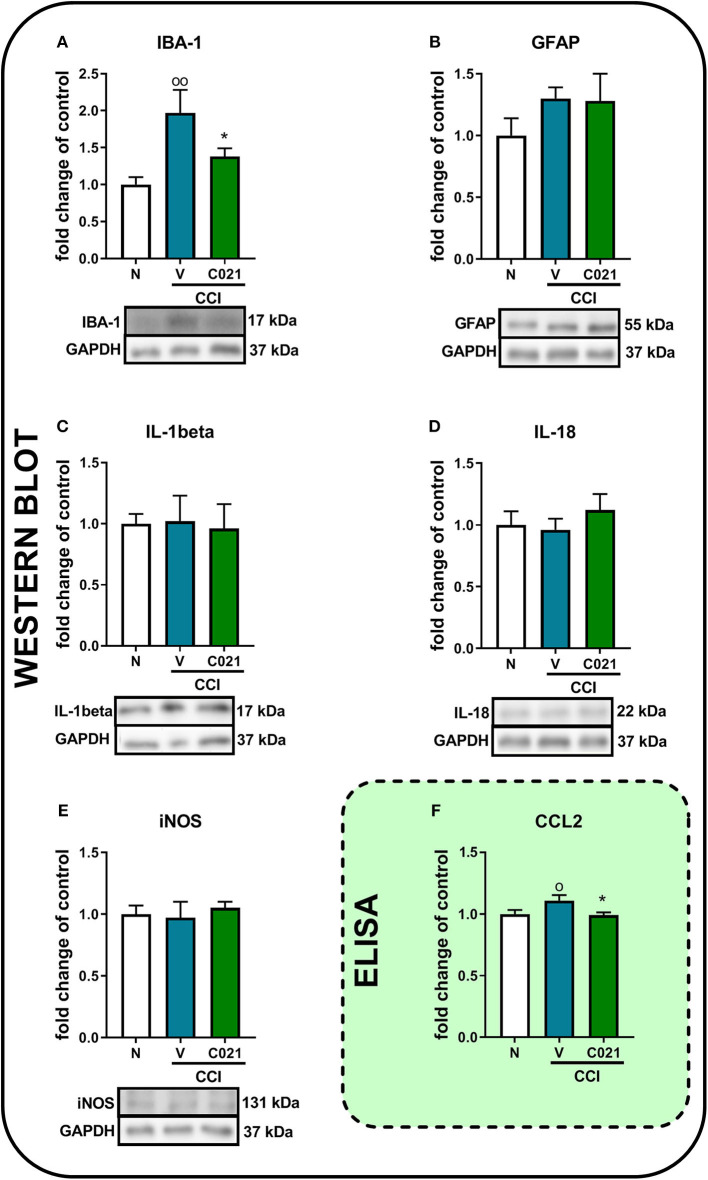
The intraperitoneal administration of C021 (a CCR4 antagonist; 5 mg/kg) influenced the spinal level of IBA-1 **(A)** and CCL2 **(F)**, but not GFAP **(B)**, IL-1beta **(C)**, IL-18 **(D)**, or iNOS **(E)**, in CCI-exposed mice, as measured by Western blotting **(A–E)** and ELISA **(F)**. Lumbar segments of the spinal cord were collected after repeated i.p. administration of C021 16 and 1 h before CCI and then twice daily for 12 days 6 h after the last administration. The data are presented as the mean ± SEM from Western blot, and the numbers of samples are as follows: N (*n* = 7–8) and CCI, V+V (*n* = 7–8), and C021+V (*n* = 7–8); and ELISA, N (*n* = 12) and CCI, V+V (*n* = 11) and C021+V (*n* = 10). The results were analyzed using one-way ANOVA with Bonferroni's multiple comparisons *post hoc* test. ^o^*p* < 0.05 and ^oo^*p* < 0.01 indicate differences between the N and V+V-treated CCI-exposed mice; *p < 0.05 indicates differences between V+V-treated and C021+V-treated CCI-exposed mice. Abbreviations: CCI, chronic constriction injury; N, naive; V, vehicle.

Pretreatment with C021 followed by the 12-day administration of C021 (twice daily, 5 mg/kg, i.p.) significantly diminished the CCI-induced enhancement of the protein level of IBA-1 compared with that in the naive animals, as measured by Western blot analysis [*F*_(2, 18)_ = 6.063, *p* = 0.0097] (Figure 8A). We did not observe changes in GFAP levels on day 12 after CCI, and C021 did not affect the level of this protein (Figure 8B). Similarly, the protein levels of IL-1beta, IL-18, and iNOS were not affected after C021 administration (Figures 8C–E). Additionally, the membranes for each Western blot analysis are presented in the Supplementary Materials (Supplementary 2). Moreover, pretreatment with C021 followed by the 12-day administration of C021 diminished the CCI-induced enhancement of the level of CCL2 in the spinal cords of CCI-exposed mice compared with that in naive mice, as measured by ELISA [*F*(_2,30_) = 3.52, *p* = 0.0424] (Figures 8F).

## Discussion

The results of our research demonstrated that a single i.t. or i.p. administration of C021, a CCR4 antagonist, dose dependently diminished neuropathic pain-related behaviors in CCI-exposed mice. After sciatic nerve injury, the spinal levels of CCL17 and CCL22 remained unchanged, unlike the level of CCL2, which was upregulated until day 14 after CCI. Importantly, our results provide evidence that CCL2 may evoke pain-related behaviors in naive mice through CCR4 because the pronociceptive effects of CCL2 were diminished by a CCR4 antagonist. In CCI-exposed mice, the pharmacological blockade of CCR4 enhanced the analgesic properties of morphine and buprenorphine, delayed the development of morphine tolerance, and improved the antinociceptive effects of chronically administered buprenorphine. Our novel findings suggest that C021 treatment prevented the increase in Iba-1 protein level and probably and, as a consequence, diminished the level of CCL2. The obtained results provide evidence that CCR4 may serve as a potential therapeutic target for neuropathic pain therapy or polytherapy.

CCR4 is expressed in the dorsal root ganglion (DRG) (38), spinal cord (39), and brain (40) by neuronal (17) and non-neuronal cells, for example, microglia, astroglia, Th2 cells, dendritic cells, macrophages, natural killers, and platelets (18, 20). Its importance in the pathogenesis of diabetes (41), multiple sclerosis (18), asthma (42), and dermatitis (43) was previously described; however, the role of CCR4 in painful neuropathy has not been studied so far. The first CCR4 ligand, CCL17 [thymus and activation-regulated chemokine (TARC)] (22) might be produced by not only thymus and peripheral blood mononuclear cells but also dendritic cells (18) and neurons (44). It has already been described that CCL17 levels are elevated in the serum of patients suffering from fibromyalgia (45) and in the cerebrospinal fluid of patients with multiple sclerosis (46) as well as in dendritic cells in an animal model of experimental autoimmune encephalomyelitis (47). Furthermore, recent studies have indicated that the neutralization of CCL17, which is elevated in the synovial fluid in a mouse model of osteoarthritis, relieves pain (48). The second CCR4 ligand, CCL22 [macrophage-derived chemokine (MDC)], which shares 37% identity at the amino acid level with CCL17 (21), is released by macrophages and serves as a chemoattractant for T lymphocytes, NK cells, monocytes, and dendritic cells (18, 49). CCL22 is considered to be upregulated in the lungs of patients with allergic asthma (42) and in skin lesions during dermatitis (43). As far as we know, there is no information about the involvement of CCL17 and CCL22 in the pathogenesis of neuropathic pain. Under physiological conditions, chemokines are constitutively expressed in nervous system, however, usually on a very low level (14, 15). In the present paper, we gave first evidence that after sciatic nerve injury in mice, the spinal expression of CCL17 and CCL22 remains unchanged compared with that of the control group. These results are in agreement with our previous study showing that in a rat model of neuropathic pain, there is a lack of spinal changes in *CCL17* and *CCL22* mRNA levels after nerve injury (35). In our pharmacological study, we provided evidence that the i.t. injection of CCL17 and CCL22 caused pain-related behaviors in naive mice, but the preinjection of a CCR4 antagonist inhibited the pronociceptive effects of these chemokines. What is more, CCL17 and CCL22 might also regulate peripheral nociceptive processes, because their mRNA level is upregulated in DRGs, 7 days after sciatic nerve injury in rats (35). Here, we showed for the first time that a single i.t. injection of C021, as well as a single i.p. injection of C021, reduced hypersensitivity in neuropathic mice. In the light of these biochemical and behavioral results, we can hypothesize that i.p. administration of the C021 is effective owing to ability to diminish the levels of pronociceptive CCL17 and CCL22 in DRG; however, this requires further study.

Based on recent reports, there are some hypotheses that CCL2 [monocyte chemoattractant protein-1 (MCP-1)] may also act through CCR4 (19). This agrees with our results showing that the i.t. preinjection of a CCR4 antagonist can block the pronociceptive properties of CCL2 in naive mice. Data in the literature provide evidence that CCL2 is produced by a variety of cell types, including fibroblasts and astrocytes, but mainly by monocytes and microglial cells (50), and serves as a chemoattractant for monocytes, T lymphocytes, and natural killer cells (51). It has been shown that this chemokine participates in the pathogenesis of various diseases, such as multiple sclerosis (51), rheumatoid arthritis (52), and insulin-resistant diabetes (53). Many studies have suggested that CCL2 plays a crucial role in neuropathic pain development at the spinal cord and DRG level (14, 25, 54). Recent studies have revealed an increase in CCL2 levels, which is associated with strong spinal activation of glial cells and, consequently, the development of neuropathic pain-related behaviors, after peripheral nerve injury in different structures of the nervous system (24, 25, 55, 56). This is in agreement with our results showing strong spinal mRNA upregulation of this chemokine from 2 to 14 days after sciatic nerve injury. Moreover, we observed alleviation of pain-related behaviors, reduced activation of IBA-1 in cells, and, likely as a consequence, decreased protein levels of CCL2 after repeated treatment with C021. Importantly, a single i.t. administration of C021, a CCR4 antagonist, diminished fully developed hypersensitivity after nerve injury, but this was not the case for RS5043930, a CCR2 antagonist (14). Therefore, we hypothesize that CCL2/CCR4 signaling at the spinal cord level might be important in nociceptive transmission. Moreover, taking into consideration our results and aforesaid literature, all three chemokines acting via CCR4, that is, CCL17, CCL22, and CCL2, seem to be important mediators in pathological nociceptive processes at the DRG level under neuropathy (14, 35). Therefore, we assume that analgesic action of C021 might be related with blockade of their pronociceptive effects in DRG, which would explain why i.p. administration is so effective.

Despite many basic and clinical studies on the mechanisms underlying neuropathic pain, this phenomenon has not been fully elucidated, and thus, commonly used analgesics are not sufficiently effective and cause many side effects (1). Opioids are used as third-line drugs for the management of chronic pain; however, their repeated and prolonged administration results in the development of tolerance, leading to a progressive reduction in their analgesic potency (1, 28, 57). It was previously suggested that heterologous desensitization between opioid and chemokine receptors might be one of the mechanisms that contribute to increasing the efficacy of opioids in neuropathic pain (11, 58). Changes in receptor conformation may have an impact on receptor activation and/or ligand binding. There is evidence showing that MOR and CCR5 can create heterodimers (59, 60). The beneficial effects of maraviroc (a CCR5 antagonist) on the effectiveness of opioids have already been shown in rats with neuropathic pain (15). This suggests that chemokine receptor antagonists may be used to support opioid therapy. Our studies showed that a single i.t. or i.p. coadministration of C021 and morphine (ligand of MOR, DOR, and KOR) or buprenorphine (ligand of MOR, DOR, KOR, and NOR) resulted in the potentiation of the analgesic properties of these opioids. To our knowledge, there is no evidence that CCR4 can create heterodimers with opioid receptors; nevertheless, further detailed investigations are required. Another important reason for the loss of the effectiveness of opioids in neuropathic pain is the activation of glial cells (6, 61). We have previously showed in both *in vivo* and *in vitro* studies that microglial inhibitor, minocycline, enhances the effectiveness of selective MOR, NOR, and KOR, but not DOR ligands under neuropathy (62, 63). The minocycline acts as an inhibitor of microglial activation; therefore, both morphine and buprenorphine effectiveness are enhanced in a CCI model. This lets us assume that C021 improves opioid effectiveness by similar mechanism—for morphine, the beneficial analgesic effect is probably related to MOR and KOR, as in case of buprenorphine also with NOR—however, further research is required. Moreover, it was previously shown that minocycline delays the development of morphine tolerance (28). Here, we observed that C021 had beneficial effects on the development of morphine tolerance. Accumulating evidence indicates that activated microglia and astrocytes release several proinflammatory factors (e.g., IL-1beta, IL-18, iNOS, and CCL2) in the spinal cord and thus increase pathological pain conditions (7, 8, 14). It has already been shown that in healthy animals, repeated morphine exposure causes the activation of microglia and results in the upregulation of key proinflammatory cytokines, which contributes to the sensitization of spinal neurons (64, 65). Based on our results showing strong elevation of IBA-1 after nerve injury and no changes in the protein levels of astroglial activation markers, microglia seems to be a more important player. Previous immunohistochemical analyses performed by our team (66) and others (67–71) visualized the changes in microglial activation in the spinal cord. It has been previously demonstrated that after peripheral nerve injury, activated microglial cells proliferate and change their morphology from ramified to amoeboid, as well as increasingly expressed markers such as IBA-1 (72). Interestingly, we did not observe any significant changes in the levels of IL-1beta, IL-18, or iNOS 12 days after nerve injury. However, it is known from our previous studies that these factors are especially crucial in the early phase of neuropathic pain development (14). On the basis of our current studies, we cannot exclude that the repeated administration of C021 influences these factors at earlier time points; thus, further experiments are needed to dispel these doubts. Moreover, recent studies have suggested that CCL2 is a key mediator of spinal microglial activation and is strictly involved in lower opioid effectiveness in naive mice (73). Furthermore, CCL2 neutralization can enhance the effectiveness of morphine in a mouse model of neuropathic pain (25). Currently, we showed that blocking CCR4 simultaneously to reduce the level of microglia/macrophage activation on the 12th day post-CCI prevents the upregulation of CCL2.

In addition to morphine, buprenorphine is widely used in the clinic for chronic pain treatment. It was recently demonstrated that the usage of CCL2 neutralizing antibodies enhances the analgesic potency of buprenorphine in neuropathic mice (25); therefore, we suggest that the potentiation of the antinociceptive effects of buprenorphine after repeated C021 treatment might also be associated with decreased levels of immune factors such as CCL2. As we mentioned before, chemokine receptors are able to form heterodimers with opioid receptors. It was previously documented that both MOR and NOR independently drive buprenorphine-induced analgesia in mice (74). In our study, we demonstrated that buprenorphine is an effective analgesic when administered for 12 days. However, after 5 days, it slightly loses its antinociceptive potential, which remains at a constant level; thus, we observed the known buprenorphine “ceiling effect” (75). The results suggest that in the early phase of neuropathy development, buprenorphine-induced analgesia is probably associated with the activation of MOR and NOR. However, in the late phase, NOR is primarily responsible for the prolonged antinociceptive effects of this opioid because MOR has already been observed to be downregulated in CCI-exposed animals (76). Our data provide evidence for the first time that CCR4 blockade increases opioid-induced analgesia and therefore could be an innovative pharmacological cotreatment designed to decrease hypersensitivity evoked by nerve injury. Additionally, our results give first evidence that C021 significantly improves the motor functions in neuropathic rats. In 2007, we have shown that minocycline not only diminished neuropathic pain-related behaviors but also improved the motor efficiency, which was associated with silencing spinal microglial activation (34). It was previously demonstrated that after peripheral nerve injury, not only sensory neurons but also motoneurons are damaged, which in turn implies strong activation of microglial cells in both the dorsal and ventral horns of the spinal cord (71). Our current results indicate that blockade of CCR4 by C021 beneficially influences spinal microglial cell activation and probably as a consequence improves the locomotor activity. Moreover, relieving the pain through C021 administration certainly allows animals to move freely, which can be also useful in accelerating regeneration (77, 78).

In light of subsequent research, we propose that CCR4 is a promising potential target for the pharmacotherapy of painful neuropathy. Our data showed that C021 diminished tactile and thermal hypersensitivity, improved motor function, and also favorably impacted the effectiveness of opioids in a mouse model of neuropathic pain. Because neuroimmune interactions are important in the development of neuropathy and considering the promising results obtained in our biochemical studies, we suggest that the pharmacological blockade of CCR4 may represent a new strategy for neuropathic pain polytherapy in the future.

## Data Availability Statement

The datasets generated for this study are available on request to the corresponding author.

## Ethics Statement

The study protocol was approved by the II Local Ethics Committee branch of the National Ethics Committee for Experiments on Animals based at the Maj Institute of Pharmacology, Polish Academy of Sciences (Krakow, Poland, LKE 75/2017,1277/2015).

## Author Contributions

JB and JM planned the study. JB, KC, KP, KK, JD, AP-M, and JM made the experiments, analyzed and interpreted the results, drafted the manuscript, and accepted the finalized version. All authors have made substantial contributions to the conception and design of the study and analysis and interpretation of data for the present study, gave final approval of the version to be published, and agreed to be accountable for all aspects of the research in ensuring that questions related to the accuracy or integrity of any part of the study are appropriately investigated and resolved.

## Conflict of Interest

The authors declare that the research was conducted in the absence of any commercial or financial relationships that could be construed as a potential conflict of interest.
