# Supplementary material for: CCR4 Antagonist (C021) Administration Diminishes Hypersensitivity and Enhances the Analgesic Potency of Morphine and Buprenorphine in a Mouse Model of Neuropathic Pain
Source: Front Immunol. 2020 Jul 14;11:1241. doi: 10.3389/fimmu.2020.01241 (PMC7372009; doi:10.3389/fimmu.2020.01241)
Supplement: Supplementary file 1 [file Image_1.pdf]

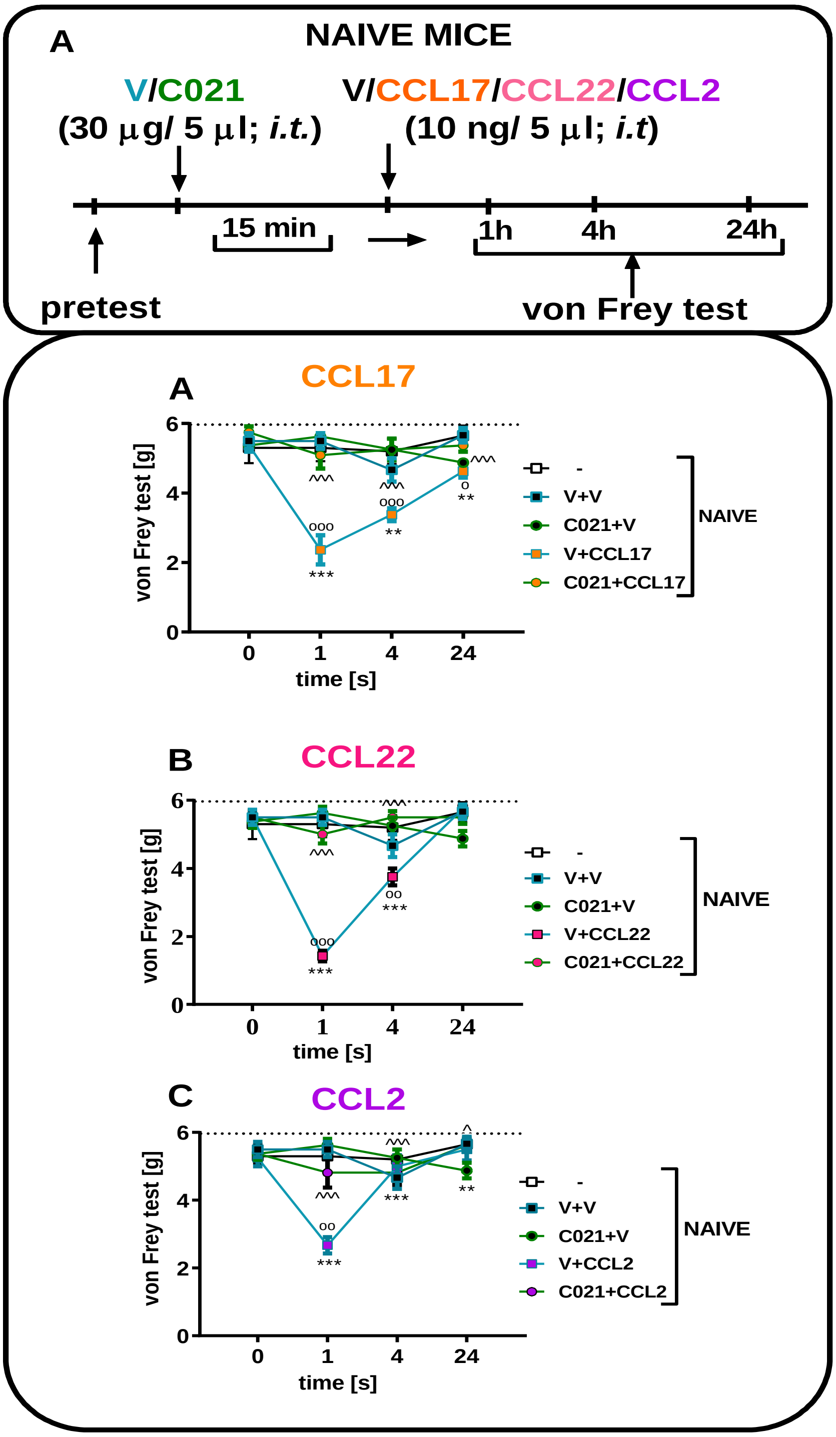

Supplementary Figure 1. The pronociceptive effect of a single intrathecal administration (10 ng/5 µl) of CCL17 (B), CCL22 (C), or CCL2 (D) is diminished by intrathecal pretreatment with C021 (30 µg/5 µl) in naive mice. Behavioral tests were performed 1 h 15 min, 4 h 15 min and 24 h and 15 min after C021 treatment, or 1, 4 and 24 h after CCLs administration (A). Tactile hypersensitivity (von Frey test) was measured (B, C, D). The data are presented as the mean ± SEM [N: (n=5); CCI: V+V (n=6), V+CCL17 (n=8), V+CCL22 (n=8), V+CCL2 (n=7), C021+V (n=8), C021+CCL17 (n=8), C021+CCL22 (n=8), C021+CCL2 (n=7-8)], and the results were evaluated by one-way ANOVA followed by Bonferroni's multiple comparisons post hoc test of selected pairs at respective time points. *p<0.05; **p<0.01; ***p < 0.001 indicates differences between the V+V- and V + CCL17/CCL22/CCL2-treated naive mice; ^p < 0.05 ^^^p < 0.001 indicates differences between the V+CCL17/CCL22/CCL2- and C021+CCL17/CCL22/CCL2-treated naive mice; ^{o}<0.05; ^{oo}<0.01; ^{ooo}<0.0001 indicates differences compared with naive mice without any treatment. The dotted lines indicate the cut off value of the test. **Abbreviations: C021**, C021 dihydrochloride; **N**, naive; **V**, vehicle.
