# Supplementary material for: CCR4 Antagonist (C021) Administration Diminishes Hypersensitivity and Enhances the Analgesic Potency of Morphine and Buprenorphine in a Mouse Model of Neuropathic Pain
Source: Front Immunol. 2020 Jul 14;11:1241. doi: 10.3389/fimmu.2020.01241 (PMC7372009; doi:10.3389/fimmu.2020.01241)
Supplement: Supplementary file 2 [file Presentation_1.pdf]

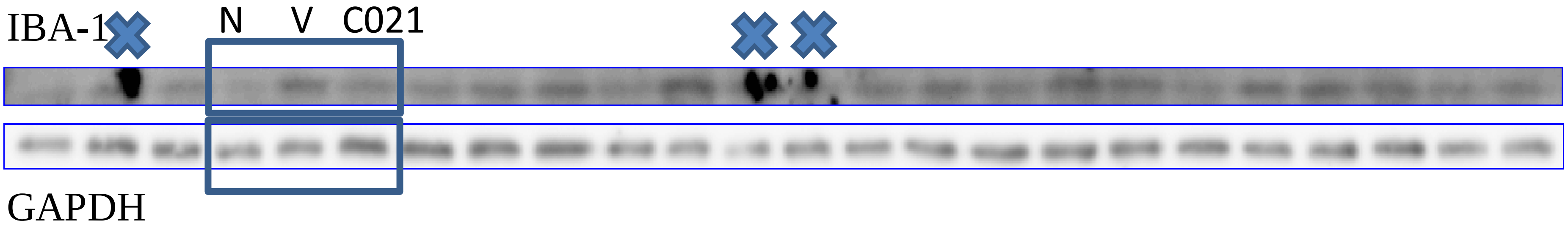
IBA-1
N
V
C021
GAPDH

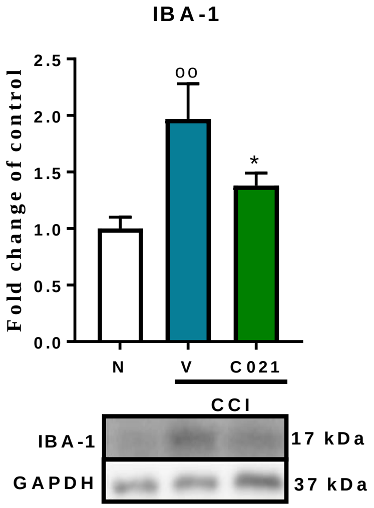
IBA-1
Fold change of control
2.5
2.0
1.5
1.0
0.5
0.0
oo
*
N
V
C021
CCI
IBA-1
GAPDH
17 kDa
37 kDa

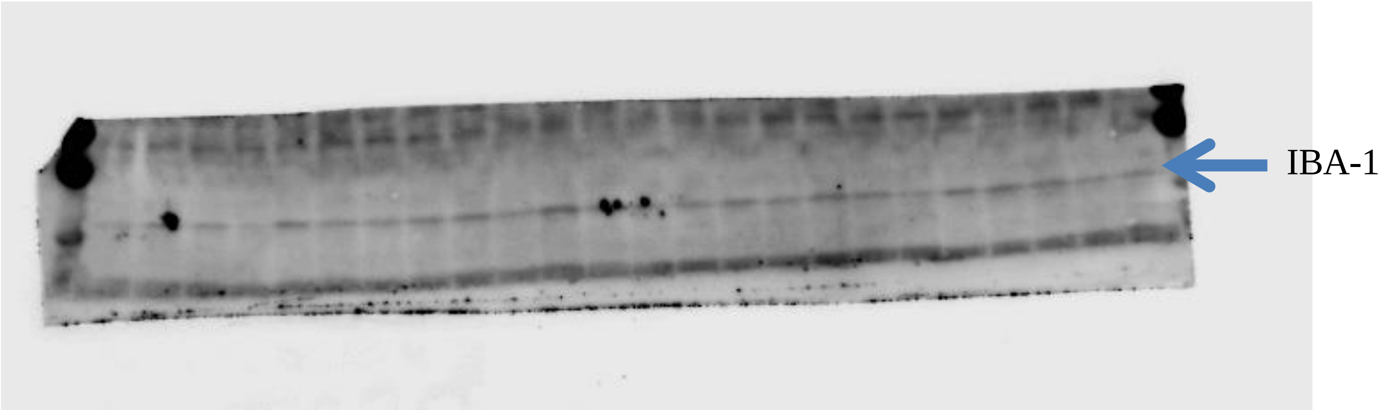
IBA-1

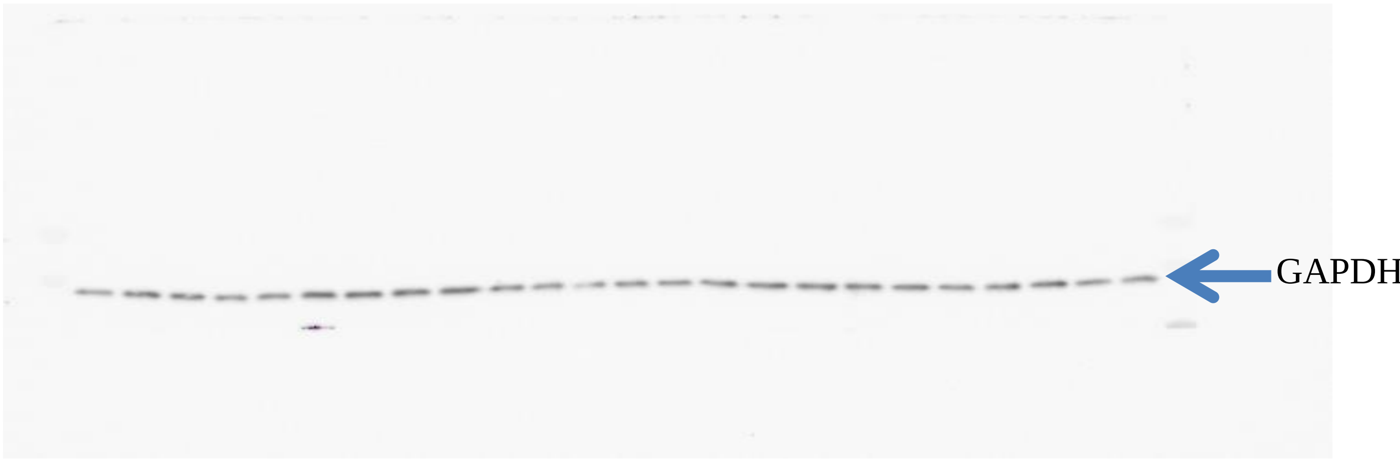
GAPDH

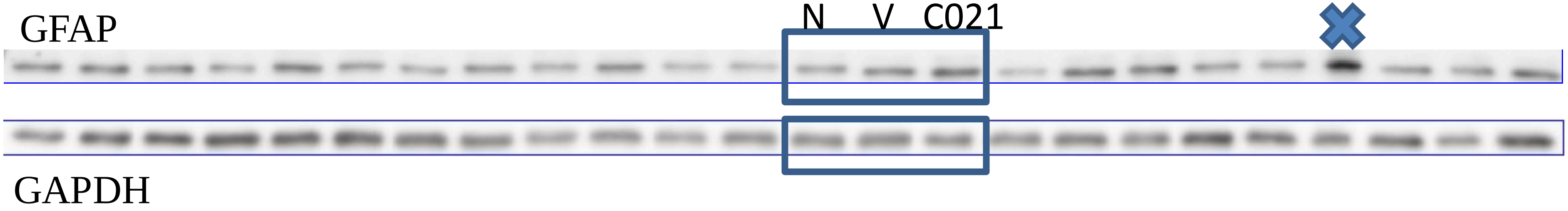
GFAP
N
V
C021
GAPDH

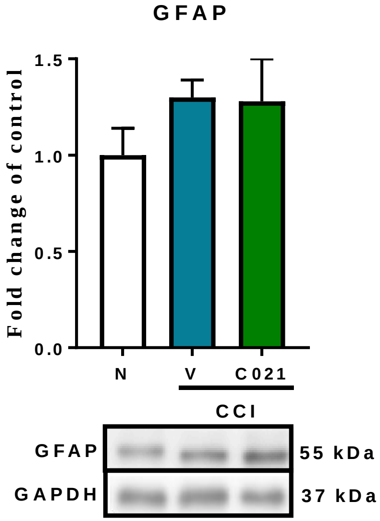
GFAP
Fold change of control
1.5
1.0
0.5
0.0
N
V
C021
CCI
GFAP
GAPDH
55 kDa
37 kDa

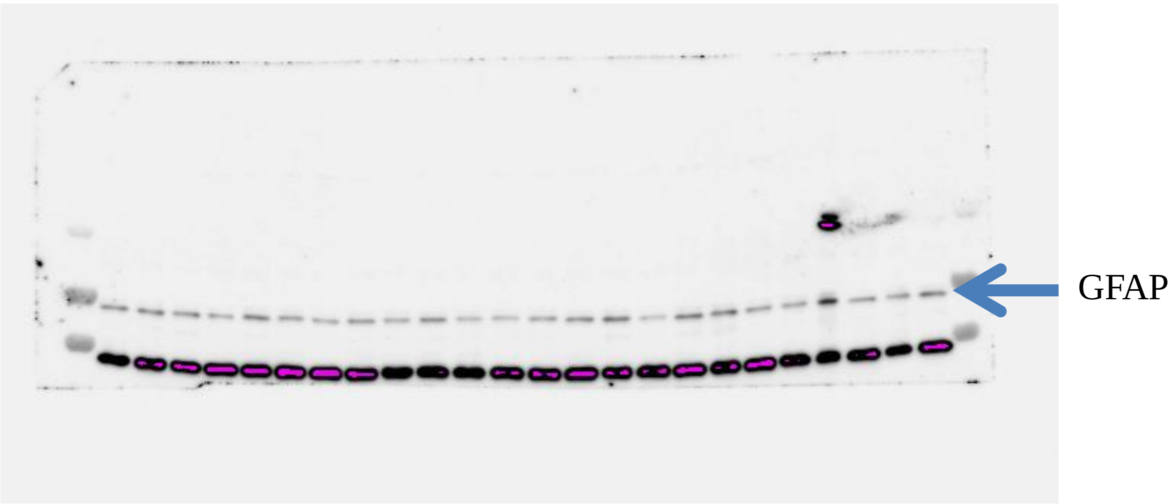
GFAP

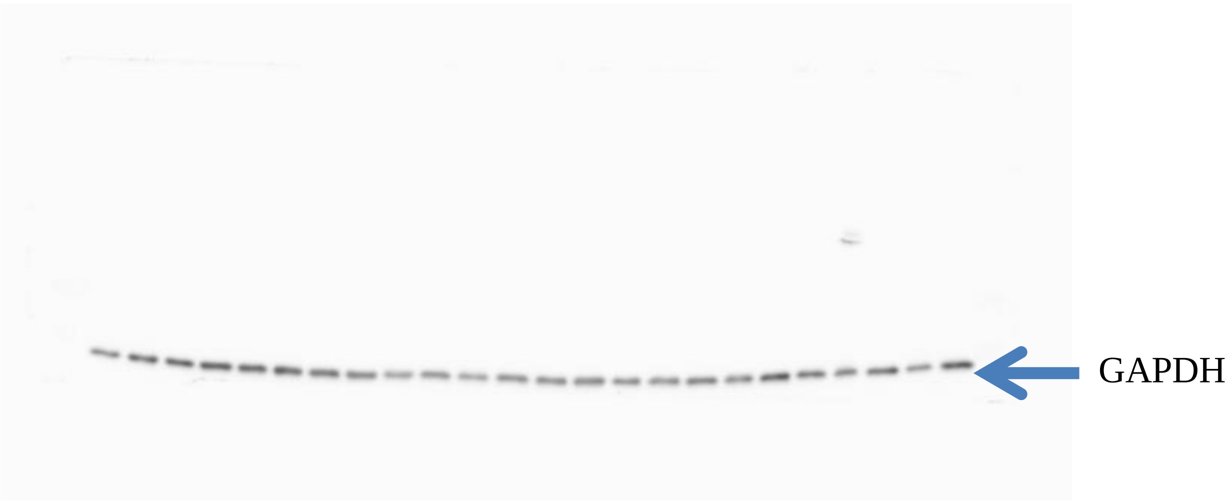
GAPDH

IL-1BETA

N V C021

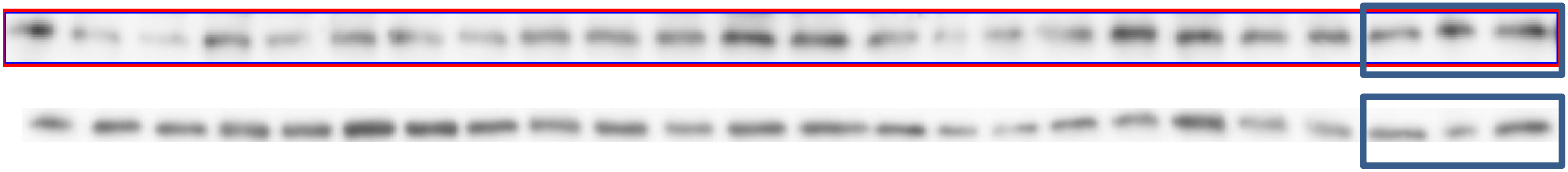

GAPDH

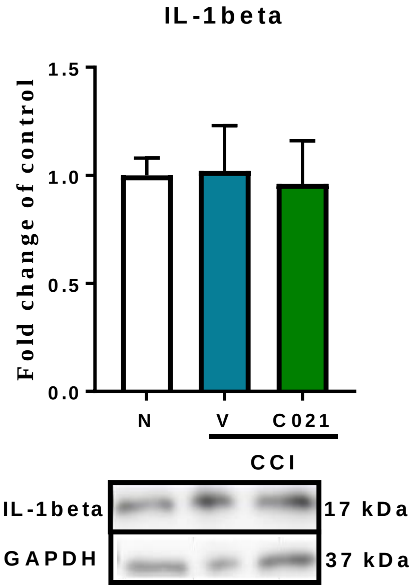

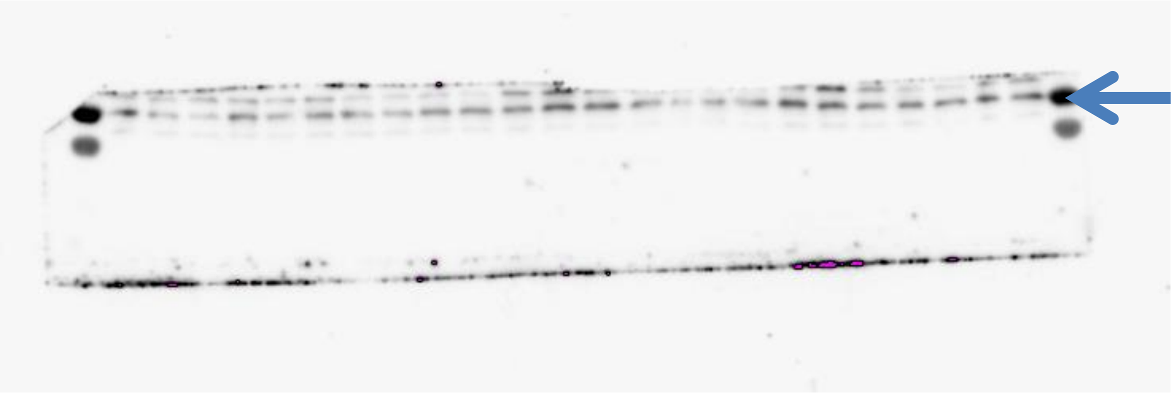

IL-1BETA

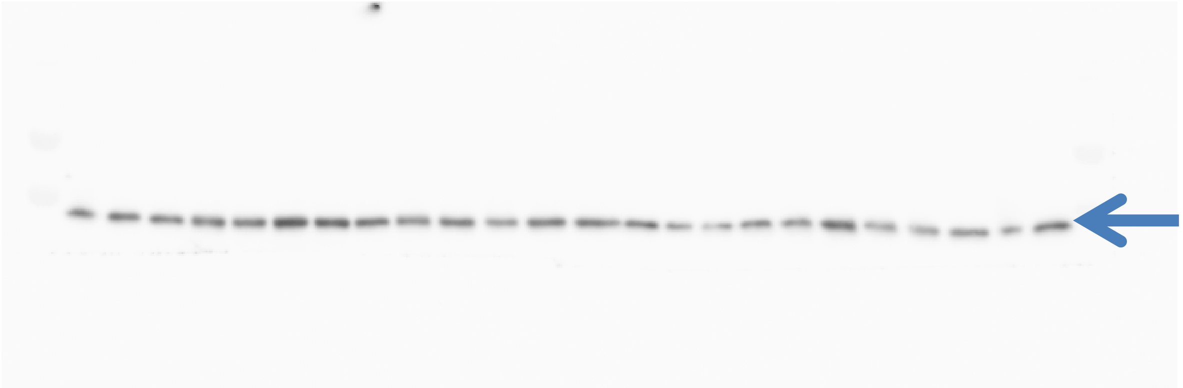

GAPDH

IL-18

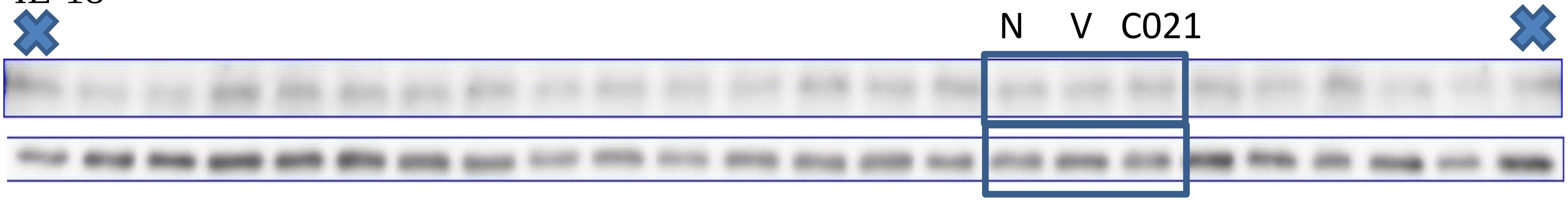
N
V
C021

GAPDH

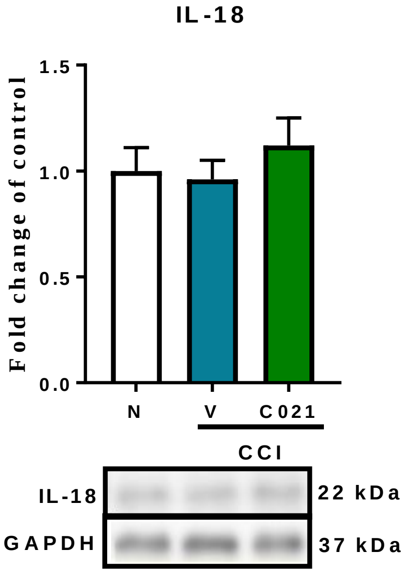
IL-18
Fold change of control
1.5
1.0
0.5
0.0
N
V
C021
CCI
IL-18
GAPDH
22 kDa
37 kDa

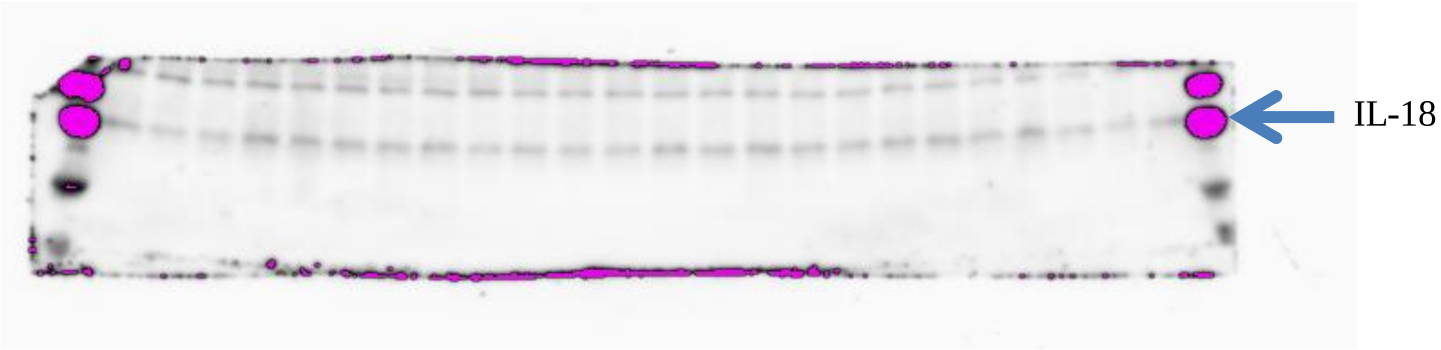
IL-18

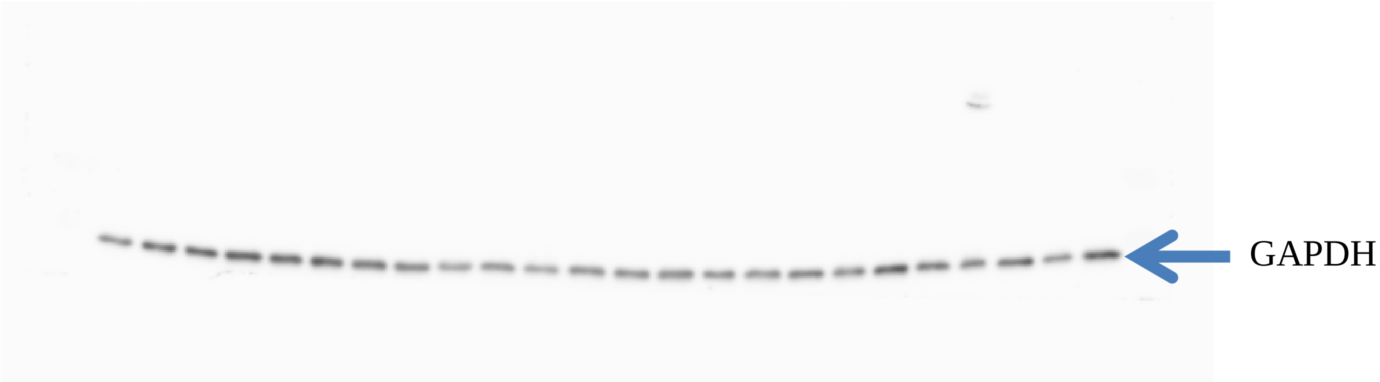
GAPDH

iNOS

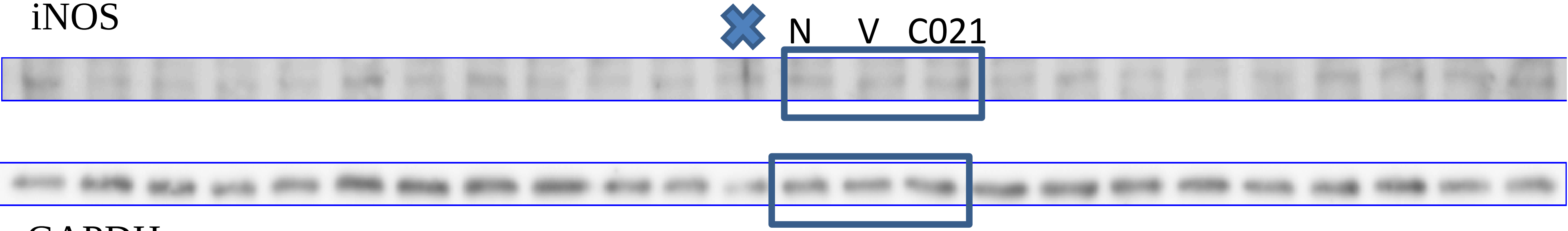

GAPDH

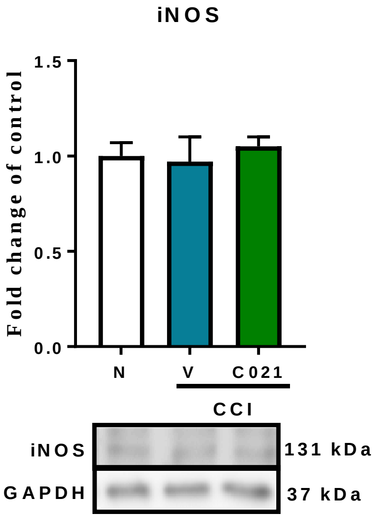

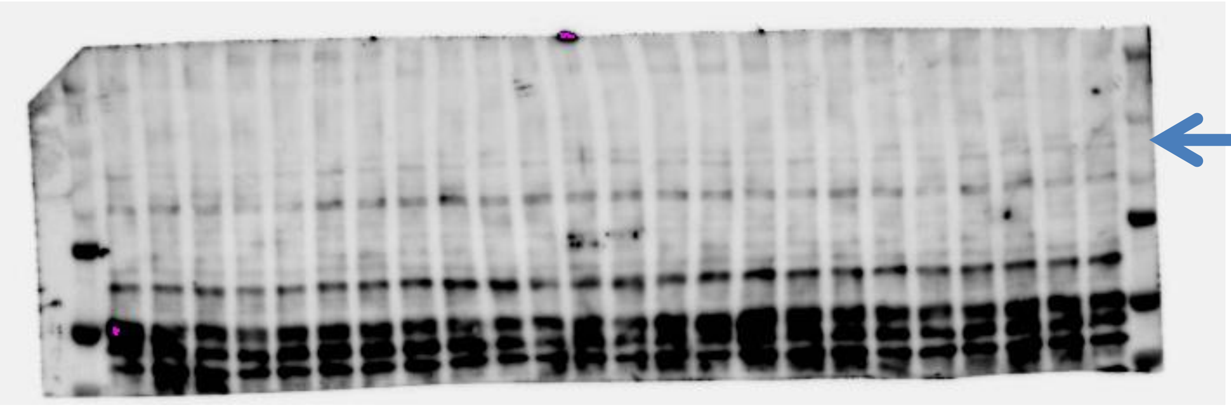

iNOS

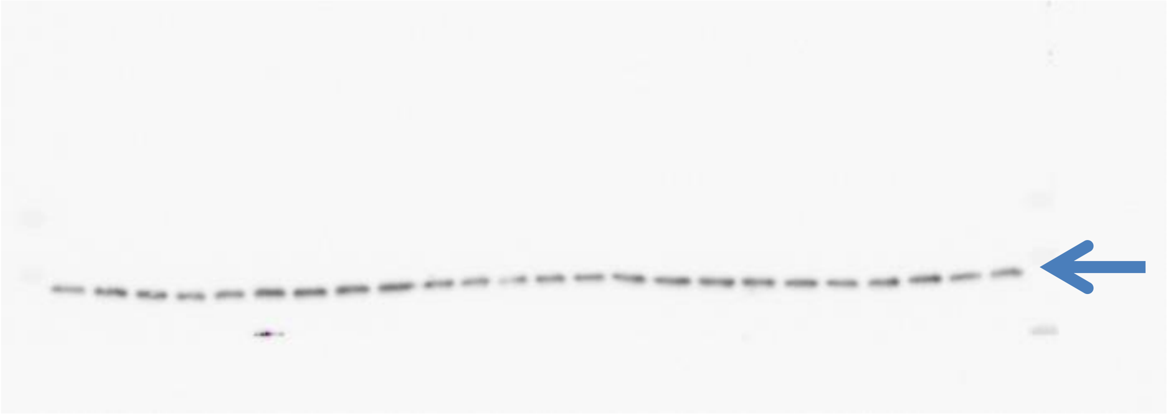

GAPDH
